# Frontal Top-Down Signals Increase Coupling of Auditory Low-Frequency Oscillations to Continuous Speech in Human Listeners

**DOI:** 10.1016/j.cub.2015.04.049

**Published:** 2015-06-15

**Authors:** Hyojin Park, Robin A.A. Ince, Philippe G. Schyns, Gregor Thut, Joachim Gross

**Affiliations:** 1 Institute of Neuroscience and Psychology, University of Glasgow, Glasgow G12 8QB, UK

## Abstract

Humans show a remarkable ability to understand continuous speech even under adverse listening conditions. This ability critically relies on dynamically updated predictions of incoming sensory information, but exactly how top-down predictions improve speech processing is still unclear. Brain oscillations are a likely mechanism for these top-down predictions [1, 2]. Quasi-rhythmic components in speech are known to entrain low-frequency oscillations in auditory areas [3, 4], and this entrainment increases with intelligibility [5]. We hypothesize that top-down signals from frontal brain areas causally modulate the phase of brain oscillations in auditory cortex. We use magnetoencephalography (MEG) to monitor brain oscillations in 22 participants during continuous speech perception. We characterize prominent spectral components of speech-brain coupling in auditory cortex and use causal connectivity analysis (transfer entropy) to identify the top-down signals driving this coupling more strongly during intelligible speech than during unintelligible speech. We report three main findings. First, frontal and motor cortices significantly modulate the phase of speech-coupled low-frequency oscillations in auditory cortex, and this effect depends on intelligibility of speech. Second, top-down signals are significantly stronger for left auditory cortex than for right auditory cortex. Third, speech-auditory cortex coupling is enhanced as a function of stronger top-down signals. Together, our results suggest that low-frequency brain oscillations play a role in implementing predictive top-down control during continuous speech perception and that top-down control is largely directed at left auditory cortex. This suggests a close relationship between (left-lateralized) speech production areas and the implementation of top-down control in continuous speech perception.

## Highlights

• Frontal top-down signals modulate low-frequency oscillations in auditory cortex
• Top-down signals are stronger for left auditory cortex than for right auditory cortex
• Speech-auditory cortex coupling is enhanced as a function of top-down signals

## Teaser

Park et al. provide new insights into the role of low-frequency oscillations in top-down control during continuous speech processing. They demonstrate causal top-down signals from frontal and motor areas largely directed at left auditory cortex. Speech-auditory cortex coupling is enhanced as a function of stronger top-down signals.

## Results

**Figure 1:**
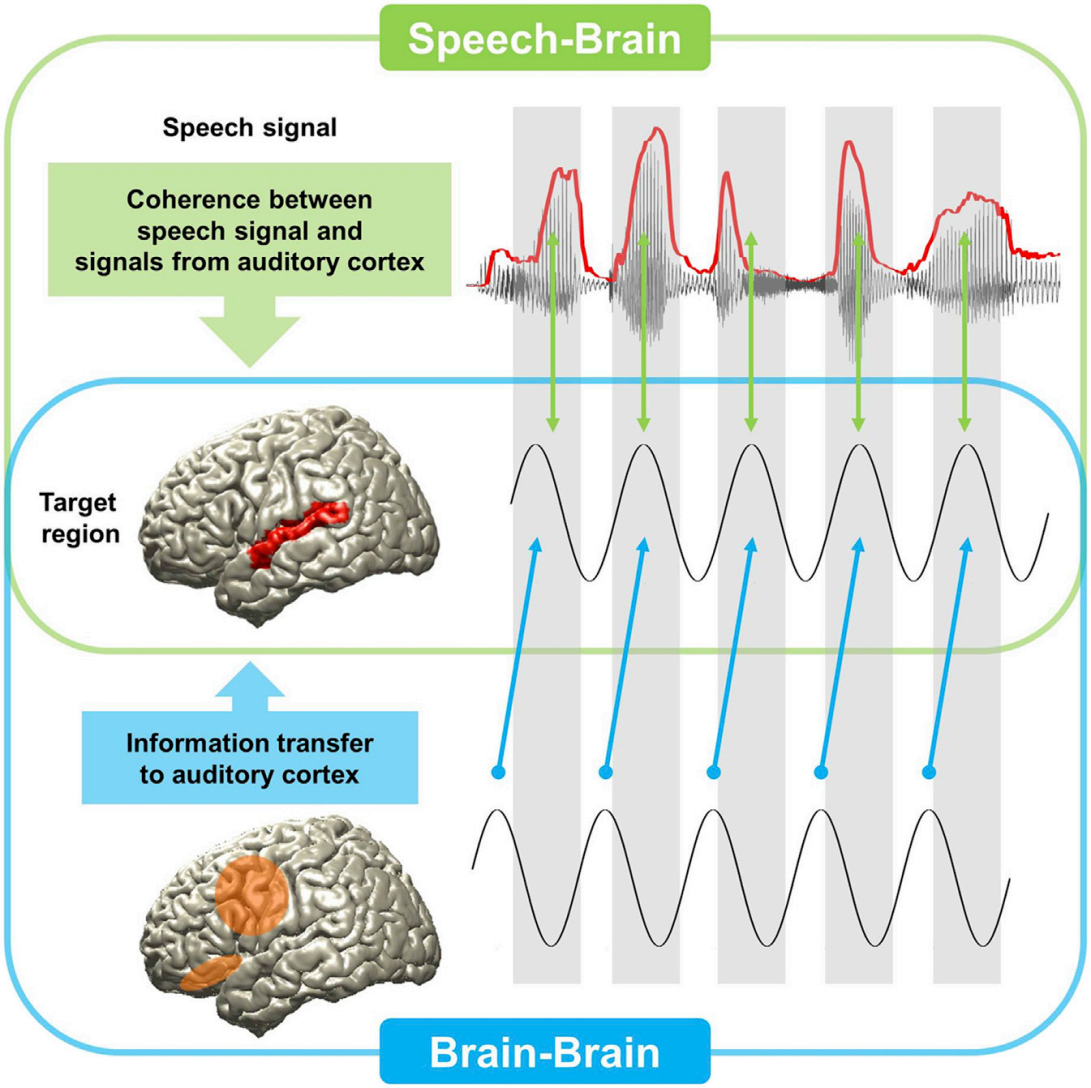
A Schematic Figure of Speech-Brain and Brain-Brain Couplings Speech-brain coupling (green box): quasi-rhythmic components in speech are known to entrain low-frequency oscillations in auditory areas. This entrainment is evident as coherence between speech envelope and neural activity in auditory cortex (target region). Brain-brain coupling (blue box): we hypothesize that low-frequency oscillations in auditory cortex (target region) are modulated by top-down signals from higher-order areas, thereby changing the gating of speech input.

An important aim of our analyses is to test the functional hypothesis that higher-order brain regions influence auditory cortices in a top-down manner to improve the alignment of auditory oscillations with the quasi-rhythmic components of speech (schematically illustrated in Figure 1). We develop our analysis in three steps. First, we demonstrate a top-down directional causal influence of higher-order regions on auditory cortices. Second, we show that this causal influence is primarily lateralized to left auditory cortex. Finally, we show that the functional role of the top-down influence is to improve the speech-brain rhythmic phase alignment.

**Figure 2:**
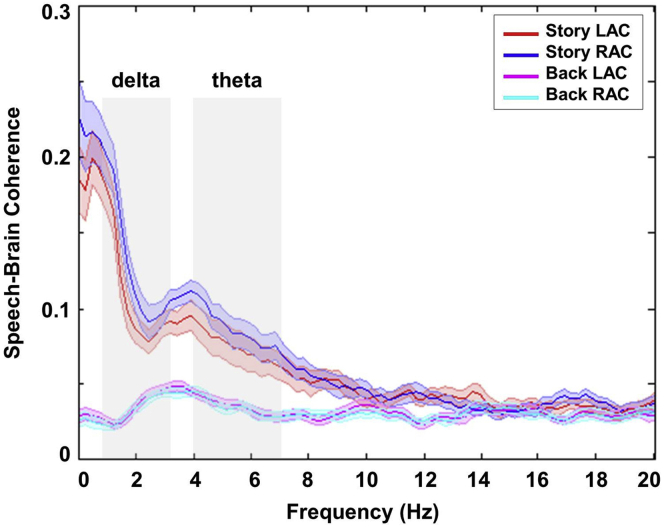
Speech-Brain Coupling Coherence between speech envelope and low-frequency oscillations (1–20 Hz) in the left auditory cortex (LAC) and right auditory cortex (RAC) in the intelligible speech (story) and unintelligible speech (back) conditions. Low-frequency brain oscillations (delta: 1–3 Hz band; theta: 4–7 Hz band) are entrained by the speech envelope in the intelligible speech (story) condition.

Our results are based on directional connectivity analysis using transfer entropy (TE). TE is an information theoretic measure that quantifies directed causal effects between time series. We focused our analysis on the phase of low-frequency brain oscillations (delta: 1–3 Hz band; theta: 4–7 Hz band) because they correspond to prosody and syllable rate [3], and they are robustly entrained by continuous speech in auditory cortex (Figure 2, reproducing [4]). This analysis produced volumetric, whole-brain maps where each voxel value represents the strength of directional (top-down) connectivity from this voxel to the left and right auditory cortices, respectively. We computed these two volumetric maps for all 22 participants, two frequency bands (delta, theta), and two experimental conditions (intelligible [story] and unintelligible [back] speech). In the story condition, participants listened to a 7-min-long continuous story. In the back condition, the same story was played backward (see Supplemental Experimental Procedures for details).

**Figure 3:**
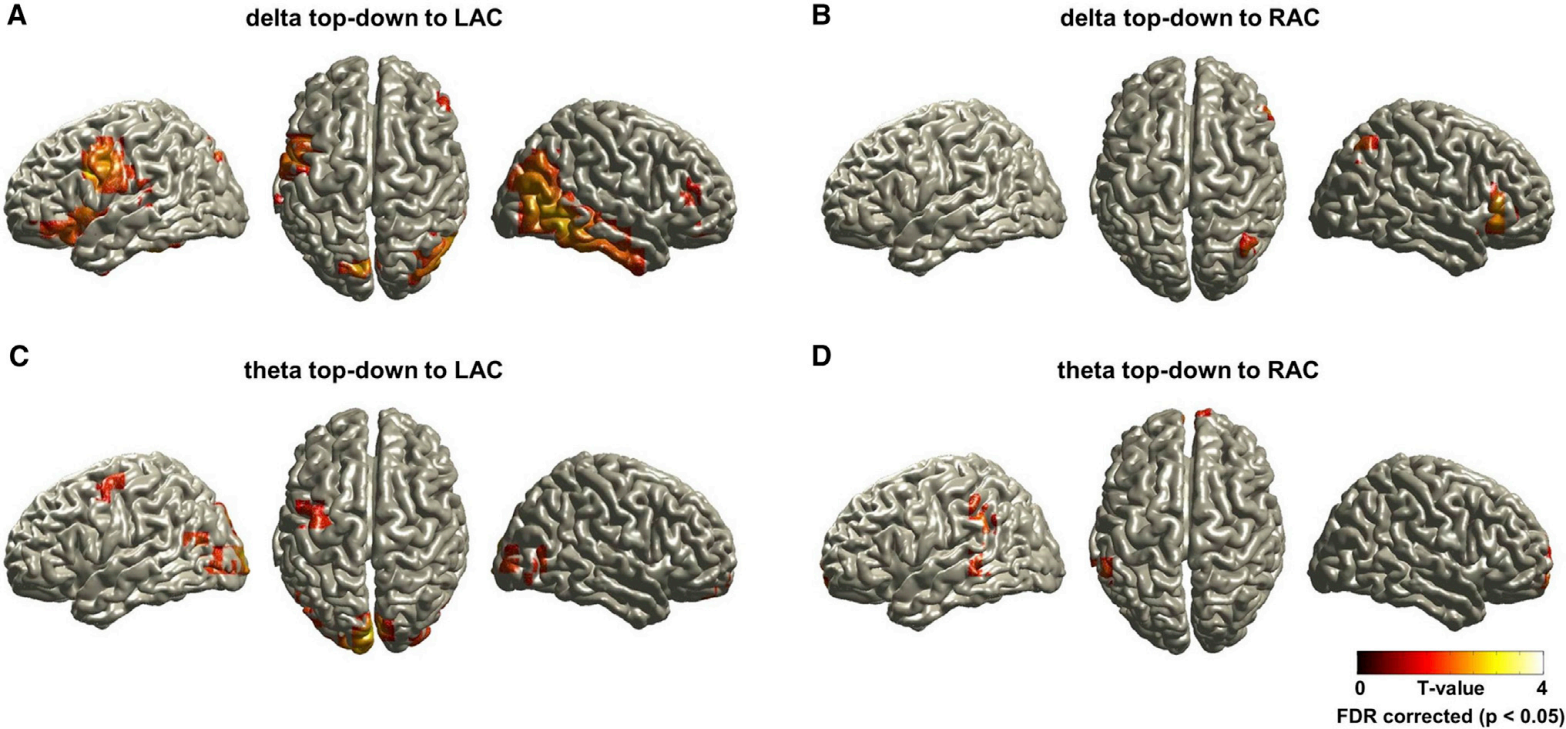
Volumetric Maps of Top-Down TE on Auditory Phase for Delta and Theta Oscillations Transfer entropy (TE) from each voxel to reference voxels in the LAC and RAC was computed in each condition (story and back), then statistically compared between conditions (p < 0.05, corrected for multiple comparisons using FDR). Areas with significantly increased TE in the story condition are shown in (A) delta top-down to LAC, (B) delta top-down to RAC, (C) theta top-down to LAC, and (D) theta top-down to RAC (see Figure S1 for back > story). (A and C) An extensive network of areas showed increased TE in the story compared to the back condition to LAC. For the delta band (A), the network comprised right middle and inferior temporal gyri, left superior parietal lobule, L-IFG including BA 44, 45, and 47 regions extending to precentral gyrus (BA 6), and right middle and inferior frontal gyri. For the theta band (C), top-down effects on LAC originated in left cuneus, right middle temporal gyrus (BA 37), and left precentral gyrus (BA 4/6). (B and D) In contrast, only very few areas showed increased TE to RAC between conditions for both delta (B) and theta (D) bands.

First, we identified the brain areas that modulate in a top-down manner the dynamics of auditory phase. We performed false discovery rate (FDR)-corrected group statistics to reveal areas that causally change the phase of auditory delta or theta oscillations significantly more strongly in the story condition than in the back condition (see Figure S1 for opposite contrast, back > story). Figure 3 shows statistical maps of significant TE differences between story and back condition for left and right auditory cortex for delta band (upper panel) and theta band (lower panel). An extensive network of areas showed increased TE in the story condition compared to the back condition to the left auditory cortex (Figures 3A and 3C). For the delta band, these networks comprised right, middle, and inferior temporal gyri, left superior parietal lobule, left inferior frontal gyrus (L-IFG), including Brodmann area (BA) 44, 45, and 47 regions extending to precentral gyrus (BA 6), and right middle and inferior frontal gyri. For the theta band, top-down effects on the left auditory cortex originated in left cuneus, right middle temporal gyrus (BA 37), and left precentral gyrus (BA 4/6). In contrast, only few areas showed increased TE to the right auditory cortex between story condition and back condition (Figures 3B and 3D). For the delta band, these areas were located in right inferior frontal gyrus and inferior parietal lobule. For the theta band, the right auditory cortex received inputs from areas in right medial frontal gyrus, left inferior parietal lobule, and left middle temporal gyrus. Brain areas and their Montreal Neurological Institute (MNI) coordinates for Figure 3 are shown in Table S1.

The results so far demonstrate that there are higher-order areas whose causal influence on the phase of auditory areas is more pronounced during an intelligible story rather than during an unintelligible story and that this causal influence targets predominantly the left auditory cortex as shown in Figure 3.

**Figure 4:**
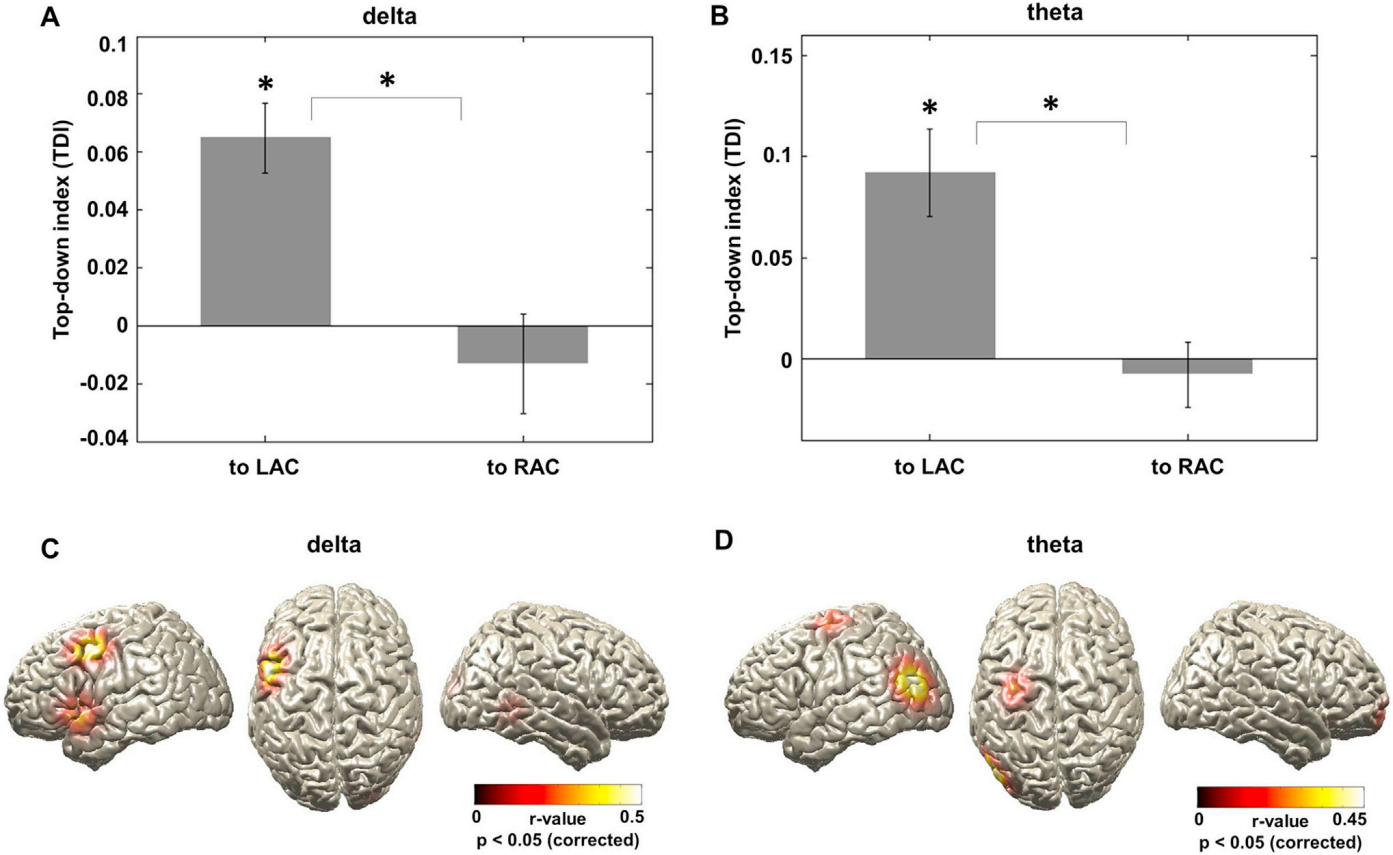
TDI for LAC and RAC and Its Correlation with Differential Speech-Brain Coupling (A and B) A normalized top-down index (TDI; (TE(story) − TE(back))/(TE(story) + TE(back))) that quantifies the degree to which higher-order brain areas differentially change the phase of LAC and RAC between conditions was computed for each participant. The mean TDI over significant voxels (and SEM) is shown for delta (A) and theta (B) frequency bands. (C and D) Correlation between TDI and differential speech-brain entrainment. TDI and differential speech-brain coherence (story − back) was correlated across subjects for delta (C) and theta (D) bands (p < 0.05, corrected). Results for delta band show significant positive correlations strongest in left frontal and precentral gyri, indicating that more top-down effects lead to better speech-brain entrainment. Results for theta band also show significant positive correlations with speech-brain coherence in left precentral gyrus and posterior temporal areas.

Next, we statistically quantified this apparent hemispheric asymmetry. For each participant, we computed a normalized top-down index (TDI) that quantifies the degree to which the identified brain areas (see Figure 3) causally change the phase of left and right auditory cortex differentially for story and back condition. TDI is defined as (TE(story) − TE(back))/(TE(story) + TE(back)). Figure 4 shows the mean TDI for the left and right auditory cortices for the delta (Figure 4A) and theta (Figure 4B) frequency bands computed from the significant areas displayed in Figure 3. TDI was significantly larger than zero for the left auditory cortex in both frequency bands (delta: t = 4.96, p < 0.001; theta: t = 4.33, p < 0.001) but not significant for the right auditory cortex (both p > 0.05).

In addition, we specifically tested for lateralization of top-down signals to the left versus the right auditory cortex. We statistically compared the mean TDI value across significant voxels (from Figure 3) to the left versus the right auditory cortex. Mean TDI was significantly higher for the left as compared to the right auditory cortex in both the delta (t = 3.72, p < 0.001) and theta (t = 3.58, p < 0.001) frequency bands.

These results demonstrate that specific higher-order areas exert a causal top-down effect on low-frequency oscillations in the auditory cortex that is stronger for intelligible speech as compared to unintelligible speech. In addition, we found a significant lateralization of these top-down effects to the left auditory cortex as compared to the right auditory cortex.

Next, we performed further analysis to better characterize top-down signals to left auditory cortex. Delay-specific TDI for left auditory cortex demonstrated that delta TDI is strongest at delays of about 50–60 ms (Figure S2A), whereas theta TDI demonstrates a cyclic modulation at multiple delays (separated by about 40 ms; Figure S2B). We also computed TE time-resolved and centered on “edges” in the continuous speech (following the approach in [4]). Both delta TE and theta TE show increases before and around edge onset (Figures S2C–S2E) in left inferior frontal and precentral gyri.

We further hypothesized that the functional role of the increased top-down effect to the left auditory cortex for the story condition (compared to the back condition) is to increase speech-brain entrainment. This functional hypothesis is based on the notion that predictions about the upcoming speech input will improve the alignment of auditory oscillations to the quasi-rhythmic speech components (such as syllables). To test this hypothesis, we correlated the TDI and differential speech-brain coherence (story − back) across participants. We computed correlations separately for each voxel (from Figure 3) and for delta (Figure 4C) and theta (Figure 4D) bands for TDI to left auditory cortex (threshold at p < 0.05, corrected). This analysis revealed significant positive correlations for the delta band that are strongest in left frontal and precentral gyri, indicating that more top-down effects lead to better speech-brain entrainment (Figures 4C, S3A, and S3B). Similarly, for the theta band, the analysis also revealed significant positive correlations with differential speech-brain coherence in left precentral gyrus and posterior temporal areas (Figures 4D, S3C, and S3D).

## Discussion

Here, we provide the first direct evidence that top-down signals during speech perception modulate the phase of low-frequency oscillations in the auditory cortex, particularly so in the left auditory cortex.

From a computational perspective, brain oscillations are ideal candidates for the neural implementation of top-down signals from higher-order areas to primary sensory areas [1–3]. During speech processing, they match the frequency of quasi-rhythmic components in speech (such as prosody and syllable rate), are entrained by these speech components, and represent excitability changes of neuronal populations that can be harnessed for gating information flow [6]. Indeed, we and others have recently shown that the phase of cortical oscillations is a likely mechanism for coding and segmentation of continuous speech [3, 4] and visual stimuli [7]. Further support comes from studies demonstrating at least partial spectral dissociation of bottom-up and top-down effects in high-frequency versus low-frequency oscillations, respectively [8, 9].

We observed top-down effects in delta and theta frequency bands. Both bands are functionally distinct in speech processing. Theta oscillations (4–8 Hz) are known to track syllabic rates, whereas delta oscillations (1–3 Hz) are associated with suprasegmental speech components such as intonation, prosody, and phrases [3, 10]. We find stronger top-down effects in delta band compared to theta band, possibly reflecting a preference of top-down signals for longer timescales required to extract contextual information. Similarly, an fMRI study using the same stimuli identified areas including L-IFG with higher sensitivity to longer timescales that allow extraction of contextual information [11].

Our results imply involvement of left frontal and motor areas in the generation of top-down signals, especially for the delta band. So far, most of our knowledge about likely sources of top-down effects in the context of speech perception comes from fMRI activation studies that use variations of speech intelligibility [12]. These studies point consistently to the L-IFG as a major source of top-down effects on early auditory areas based on increased activation during processing of degraded speech [13–18]. Indeed, anatomical connectivity between L-IFG and auditory cortex is well established in non-human primates [19] and humans [20], and L-IFG has been previously implicated in processes related to the access of mental representations.

Interestingly, recent research converges on the view that speech perception is predominantly bilateral (with different contributions from both hemispheres), whereas speech production is largely left lateralized [21, 22]. The left lateralization of top-down control demonstrated here therefore adds support to recent theories that link speech production and speech perception [23, 24]. This is even more plausible since the generators of top-down signals identified here are compatible with the classical speech production areas. In particular, coordinates of the left precentral gyrus identified here as a source of top-down control are in agreement with previously identified motor areas engaged in speech production [25].

Our causal connectivity analysis demonstrates that the phase of oscillations in the left auditory cortex is modulated more strongly by top-down signals than it is in the right auditory cortex. This is consistent with a recent model (PARLO) postulating lateralization of top-down control to the left hemisphere based on the context-specific modulations of the classical N400 event-related component [26]. Interestingly, the N400 component is generated by the same oscillations (delta/theta) that we have studied here directly and is typically lateralized to left hemisphere [27].

For the delay-specific TDI for theta band (Figure S2B), the periodic modulation of top-down influence at around 25 Hz (one peak every 40 ms) suggests a putative role of the beta band in top-down control, supporting and extending previous reports of a role of beta oscillation in top-down processing [9, 28]. In this study, we focused on delta and theta band because these frequencies correspond directly to prominent components in speech and show the strongest speech-brain coupling. However, the observed beta modulation warrants further investigation into possible causal effects between frequency bands. Possible top-down control from other frequency bands such as alpha, beta, and gamma could also provide an answer to the dominant peak around 50–60 ms in the delta band delay (Figure S2A). Importantly, future studies might be able to decode the content of these top-down signals.

In summary, we provide direct evidence for the role of low-frequency oscillations in top-down control during speech processing and demonstrate causal top-down signals from higher-order areas more to the left than to the right auditory cortex that improve speech-brain coupling.

## Author Contributions

J.G. and G.T. conceived and designed the experiments. H.P. and J.G. analyzed the data. R.A.A.I., P.G.S., G.T., and J.G. contributed reagents, materials, and analysis tools. All authors wrote the manuscript.

## Supplemental Information

Document S1. Supplemental Results, Supplemental Experimental Procedures, Figures S1–S3, and Table S1

Document S2. Article plus Supplemental Information
