# Supplementary material for: Frontal Top-Down Signals Increase Coupling of Auditory Low-Frequency Oscillations to Continuous Speech in Human Listeners
Source: Curr Biol. 2015 Jun 15;25(12):1649–53. doi: 10.1016/j.cub.2015.04.049 (PMC4503802; doi:10.1016/j.cub.2015.04.049)
Supplement: Document S1. Supplemental Results, Supplemental Experimental Procedures, Figures S1–S3, and Table S1 [file mmc1.pdf]

Current Biology

Supplemental Information

Frontal Top-Down Signals Increase Coupling of Auditory Low-Frequency Oscillations to Continuous Speech in Human Listeners

Hyojin Park, Robin A.A. Ince, Philippe G. Schyns, Gregor Thut, and Joachim Gross

Supplemental Figures and Legends

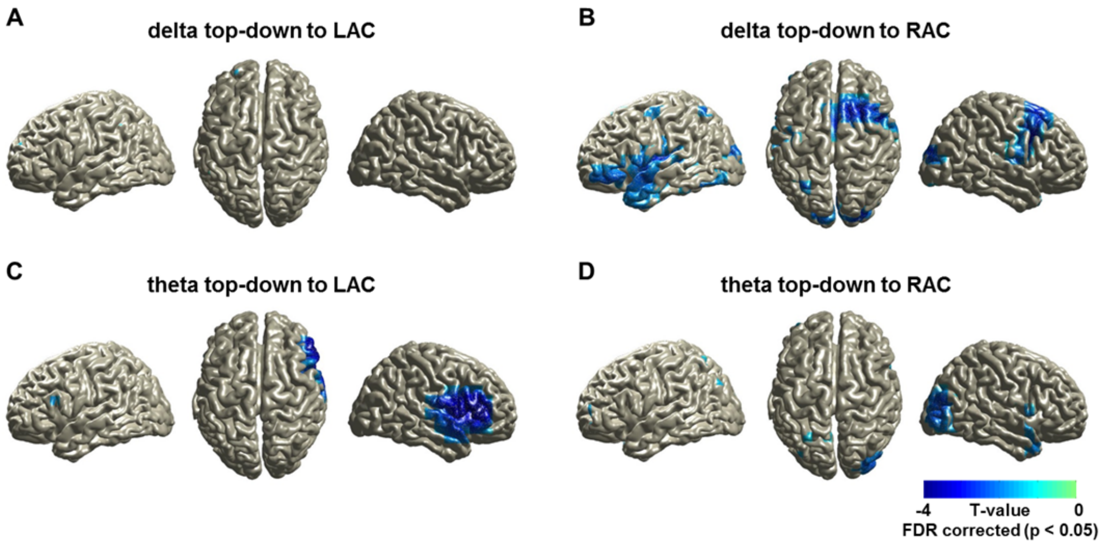

Figure S1 related to Figure 3. Transfer entropy (TE) for back condition compared to story condition

We performed group statistics on TE values for the contrast back > story condition for delta and theta bands ($p < 0.05$, FDR-corrected). Interestingly, for the delta band, top-down effects that are stronger in back compared to story condition are largely directed at right auditory cortex (Figure S1 B). In contrast, the statistical contrast story > back shows a significant lateralization towards left auditory cortex (Figure 3, 4). The most pronounced effect in the theta band is a stronger top-down effect in back compared to story condition from right temporal/frontal areas to left auditory cortex (Figure S1 C).

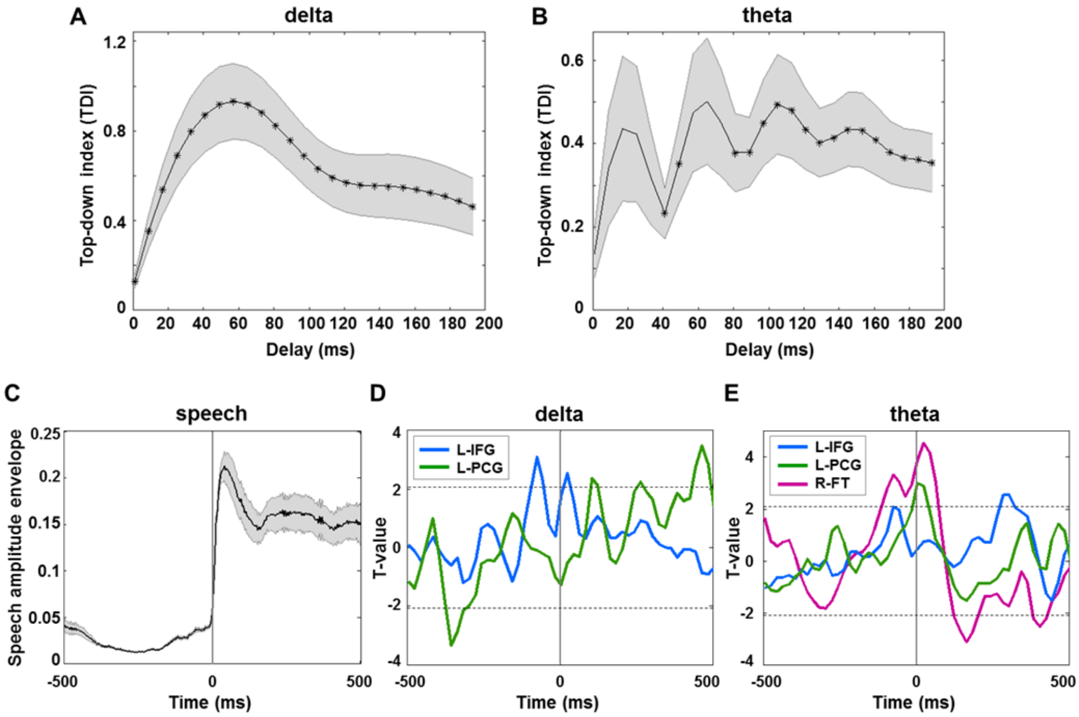

Figure S2 related to Figure 4 A and B. Top-down index (TDI) to left auditory cortex for each delay and temporal dynamics of TE time-locked to speech edges

(A and B): Top-down index (TDI) for each delay. Figure 3 shows TE averaged across 25 delays (in 8 ms steps up to 200 ms). In addition, we show here TDI to left auditory cortex for individual delays averaged over the significant voxels from story versus back condition. We compared TDI against zero at each time point (asterisk: $p < 0.05$, corrected). Delay-specific TDI for delta band (A) peaks at a delay of about 50-60 ms. This seems to be a characteristic delay where the difference between TE for story and back condition is maximal. Further investigations are required to clarify the content of this signal. Theta band (B) shows a cyclic modulation with several peaks at delays separated by about 40 ms corresponding to a frequency in the beta band. Significant differences between conditions can also be seen at the troughs of TDI modulation and seem to be caused by timing differences of TE between story and back condition.

(C, D, and E): Temporal dynamics of TE time-locked to speech edges. We have followed the approach from our previous paper [S1] and performed the transfer entropy computation in a time-resolved manner locked to the onset of edges. Edges were defined (consistent with our previous paper) as short breaks in the continuous speech followed by significant increases in the amplitude envelope. Mean and variance of time-locked speech amplitude envelope is shown in C. Time axis (0 ms; gray vertical line) is centered on the latency at the start of the rising amplitude envelope. We computed time-resolved transfer entropy (TE) to LAC (left auditory cortex) for all voxels that showed a significant condition effect as shown in Figure 3 (A, C). Across the 22 participants we computed T-values of the condition effect (story versus back) for each time point between -500 ms and 500 ms. For each brain area where the T-value was significant at $p < 0.005$ (uncorrected) for at least one time point we extracted the time series of T-values. T-value of significance level ($t = \pm 2.08$ at $p < 0.05$) is shown by horizontal dashed lines. Interestingly, different temporal pattern can be seen. In the delta frequency band (D), T-values for left IFG (L-IFG; blue) peak just before and after the onset and decrease after that (with a significantly negative slope). In contrast, T-values for left precentral gyrus (L-PCG; green) show a gradual increase (with a significant positive slope). In the theta band (E), the strongest effect is observed in right fronto-temporal areas (R-FT; purple). The T-values increase towards edge onset (reflecting significantly stronger TE for story versus back) and change sign after edge onset (reflecting significantly weaker TE for story versus back). Left IFG (L-IFG; blue) and left precentral gyrus (L-PCG; green) show strongest T-values just before or around the edge onset.

These results suggest that different areas could mediate different predictions. Those showing strongest effects around or before edge onset likely transfer predictions about the temporal onset of edges and possibly about expected syllables or phonemes although this remains speculation at this point. It should be noted that even TE values computed for time 0 ms will be based on the past 200 ms of data due to the computation of TE. Those areas showing an increase after edge onset will likely express predictions based on the incoming sensory information. For example, the difference in time between the peaks for the two regions shown in the delta band (D) suggests that the frontal region could be more involved in the prediction of upcoming speech components

(e.g. timing and content of speech onset) whereas motor region could be more involved in continuously improving predictions (after stimulus onset) based on incoming information, yet this remains speculative. Unfortunately it is difficult to track TE values for longer periods of time (e.g. seconds) because of the variability in the stimuli following an edge. Some edges will be followed by longer periods of ‘continuous’ speech whereas others might show another edge after a short period of time. In summary, this analysis suggests that different brain areas mediate different types of predictions.

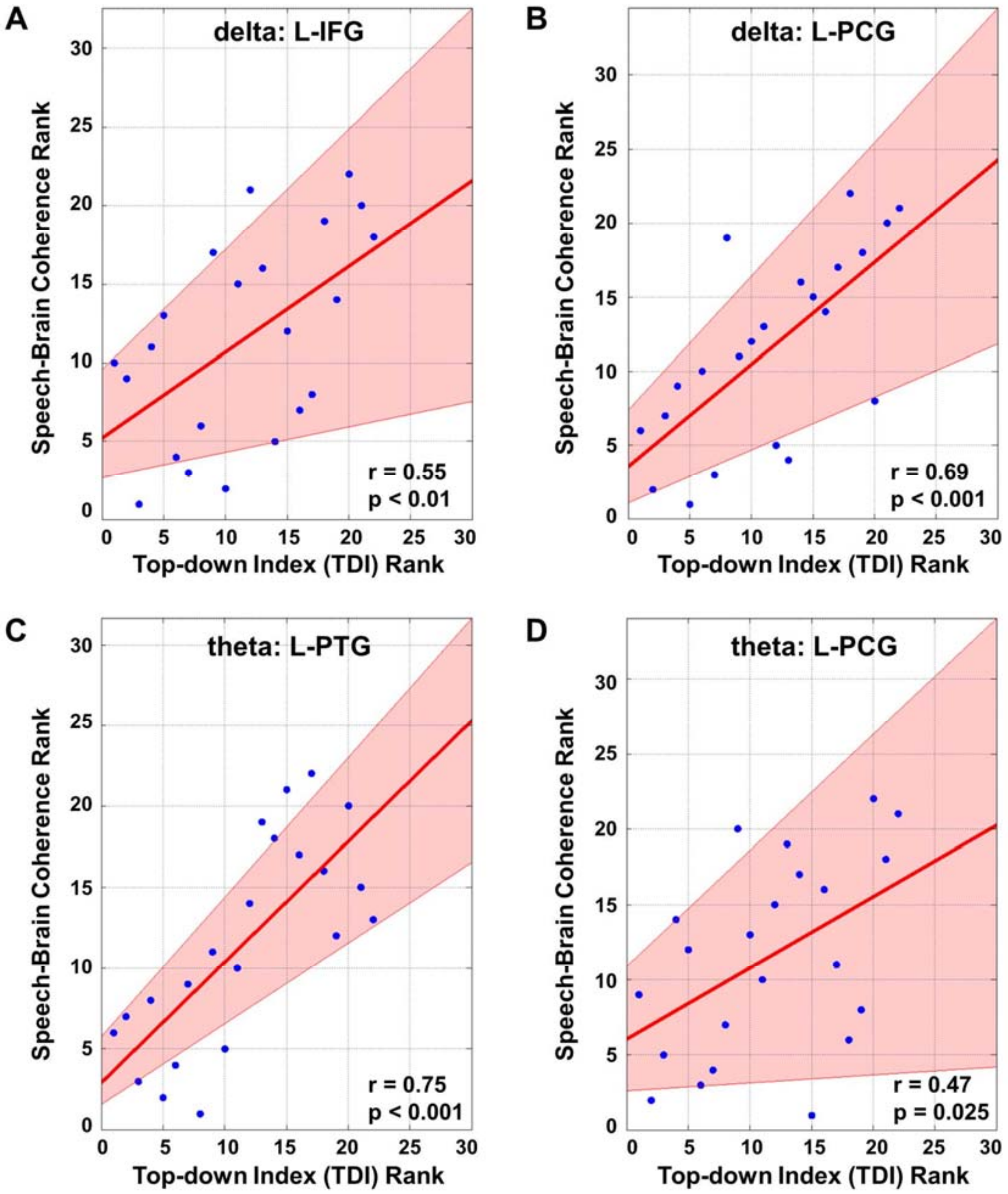

Figure S3 related to Figure 4 C and D. Correlations between top-down index (TDI) and speech-brain coupling at maximum sources

To confirm the correlations shown in Figure 4 C (delta) and D (theta), we show here the scatter plots of correlations for each significant area for delta and theta bands from Figure 4 C and D. To make sure that effects are not dominated by outliers we computed robust correlations using an

open-source toolbox [S2]. Specifically, we computed Spearman rank correlations with the 95% bootstrapped confidence interval (pink shaded areas) computed from 1000 iterations. All reported correlations are significant also based on bootstrap confidence interval.

(A) L-IFG (left inferior frontal gyrus) in the delta band: $r = 0.55$, $p < 0.01$

(B) L-PCG (left precentral gyrus) in the delta band: $r = 0.69$, $p < 0.001$

(C) L-PTG (left posterior temporal gyrus) in the theta band: $r = 0.75$, $p < 0.001$

(D) L-PCG (left precentral gyrus) in the theta band: $r = 0.47$, $p = 0.025$

Supplemental Table

Frequency band	MNI coordinates			Brain regions	L/R	BA
	**To Left Auditory Cortex (LAC)**					
	**x**	**y**	**z**			
**delta**	-52	-2	34	Inferior Frontal Gyrus (Pars Opercularis) /Precentral gyrus	L	44/6
	-42	31	-17	Inferior Frontal Gyrus	L	45/47
	-10	-80	40	Superior Parietal Lobule/Precuneus	L	7
	68	-58	-6	Middle/Inferior Temporal Gyrus	R	21
	52	39	15	Middle/Inferior Frontal Gyrus (triangular part)	R	46
**theta**	-45	-11	63	Precentral Gyrus	L	4/6
	0	-90	30	Cuneus	L	19
	60	-71	5	Middle Temporal Gyrus	R	37

Frequency band	MNI coordinates			Brain regions	L/R	BA
	**To Right Auditory Cortex (RAC)**					
	**x**	**y**	**z**			
**delta**	65	30	4	Inferior Frontal Gyrus (triangular part)	R	45
	50	-69	53	Inferior Parietal Lobule/Angular Gyrus	R	40
**theta**	-66	-44	1	Middle Temporal Gyrus	L	22
	-65	-46	36	Inferior Parietal Lobule/Supramarginal Gyrus	L	40
	5	70	-10	Medial Frontal Gyrus (orbital part)	R	11
	2	-61	-50	Cerebellum 9/Inferior Semi-Lunar Lobule	R	

Table S1 related to Figure 3. Brain areas observed in top-down transfer entropy (TE) to LAC and RAC

Brain areas and their MNI coordinates as well as Brodmann Areas (BA) for Figure 3 for both delta and theta bands are displayed.

Supplemental Experimental Procedures

Participants

22 healthy, right-handed volunteers participated in the study (11 males; age range 19–44 years, mean 27 years). All participants provided informed written consent and received monetary compensation for their participation. The study was approved by the local ethics committee (University of Glasgow, Faculty of Information and Mathematical Sciences) and conducted in conformity with the Declaration of Helsinki.

Stimulus

The main stimulus consisted of a recording of a 7-minute real-life story ("Pie-man," told by Jim O'Grady at "The Moth" storytelling event, New York). The story was presented binaurally via a sound pressure transducer through two 5 meters long plastic tubes terminating in plastic insert earpieces. Presentation was controlled with Psychtoolbox [S3] under MATLAB (MathWorks, Natick, MA). In addition to one standard presentation of the story (story condition), individuals also listened to the backward played story (back condition). Eye fixation was maintained throughout the experiment. Experimental conditions were recorded in randomized order. Stimuli have been previously used in an fMRI study [S4].

Data recording

MEG recordings were obtained with a 248-magnetometers whole-head MEG system (MAGNES 3600 WH, 4-D Neuroimaging) at 1017 Hz sampling rate. The analysis of the MEG signal was

performed using the FieldTrip toolbox [S5] (http://fieldtrip.fcdonders.nl) and in-house MATLAB code according to recently published guidelines [S6]. Another analysis of the same data has been published recently [S1].

MEG-MRI co-registration

T1-weighted structural magnetic resonance images (MRIs) of each participant were co-registered to the MEG coordinate system using a semi-automatic procedure. Anatomical landmarks (nasion, left and right pre-auricular points) were manually identified in the individual's MRI. Initial alignment of both coordinate systems was based on these three points. Subsequently, numerical optimization was achieved by using the ICP algorithm [S7]. All region-of-interest analysis for the auditory cortex is based on the mean effect of all voxels in BA 41. BA 41 voxels were the same for all participants and defined anatomically from the MNI template brain.

Source localization

Individual head models were created from anatomical MRIs using segmentation routines in FieldTrip/SPM5. Leadfield computation was based on a single shell volume conductor model [S8] using a 10 mm grid defined on the template (MNI) brain. The template grid was transformed into individual head space by linear spatial transformation. Cross-spectral density matrices were computed using Fast Fourier Transform on 1-s segments of data after applying Hanning window.

For each voxel, we computed frequency-specific spatial filters for delta (1–3 Hz) and theta (4–7 Hz) frequency bands in the dominant dipole orientation. First, we computed the covariance matrix over the full broad-band 7-minute data set to compute LCMV filters for each voxel using 7 % regularization. These time series were subjected to band-pass filtering (4th order Butterworth filter, forward and reverse). We used the SVD approach to estimate the dominant orientation

independently for each voxel. Bandpass filtered data were projected through the filter to obtain band-limited time-series for each voxel, both frequency bands (delta, theta) and both conditions (story, back). Finally, Hilbert transform was applied to compute time series of instantaneous phase.

Transfer Entropy Analysis

Transfer Entropy (TE), also known as Directed Information [S9, S10], quantifies directed statistical dependencies between two signals. Specifically, TE from signal *X* to signal *Y* quantifies to what extent knowledge of *X* reduces uncertainty in predicting the future of *Y* over and above what could be predicted from knowledge of the past of *Y* alone. TE is conceptually similar to Granger causality as it infers causal relationships from time-lagged predictability.

To determine the TE between two voxels *X* and *Y* during a particular condition (story or back), we first quantized the phase values obtained across all time points during the stimulus presentation, separately for each voxel. We used 4 bins and the bin boundaries were chosen so that the bins were equally occupied (i.e. bins represent quartiles of the distribution of phase values between $-\pi$ and π). For a specific delay *d*, we calculated TE from *X* to *Y* from the following equation:

$$
\begin{aligned}
TE_d(X \to Y) &= CMI\left(X_d ; Y \mid Y_d\right) \\
&= H(X_d, Y_d) + H(Y, Y_d) - H(X_d, Y, Y_d) - H(Y_d)
\end{aligned}
$$

Where *CMI* is conditional mutual information, *H* represents entropy and the suffix *d* represents that signal is delayed with respect to the target signal *Y* by *d* milliseconds (i.e. considers that signal *d* milliseconds prior to *Y*). We computed entropy terms from the standard formula:

$$
H(Y, Y_d) = \sum\nolimits_{a,b=1}^{4} p_{Y,Y_d}\left(a,b\right) \log_2 p_{Y,Y_d}\left(a,b\right)
$$

Where the joint distribution $p_{Y,Y_d}(a,b)$ is obtained from the multinomial maximum likelihood estimate obtained over time points:

$$p_{Y,Y_d}(a,b) = \sum_{t=d}^{Nt} \frac{\delta_a(Y(t))\delta_b(Y(t-d))}{Nt}$$

With $\delta_a(Y(t))$ a Kronecker delta function taking the value 1 if the binned phase value at *Y(t)* is quantile *a* and 0 otherwise.

No bias correction was applied since we performed statistical analysis on contrasts between conditions [S11]. For each calculation, the same number of bins was used and the same number of time points was available. To first order, the bias of mutual information depends only on these parameters [S12], so should be similar across the conditions being compared. Bias correction methods reduce bias but increase the variance of the estimator, so in this case comparisons between calculations with the same bias are better made with uncorrected estimates.

We selected all voxels in the left and right primary auditory cortices (BA 41) as reference voxels. For each reference voxel, TE was computed, as described above, from the binned instantaneous phase of all other voxels in the brain to that reference voxel. The calculation was repeated for 25 different delays, from 8 ms to 200 ms (8 ms steps).

These computations were performed for each participant, both frequency bands and both conditions (story, back). Subsequently, individual TE maps were averaged across delays and across reference voxels in the left and the right auditory cortex (BA 41) separately. Since we were interested in top-down effects on phase dynamics in the left and the right BA 41, we analyzed only the TE corresponding to causal effects from other voxels on these two reference regions. This resulted in eight TE maps per participant (two frequency bands (delta, theta), two reference regions (left and right auditory cortices) and two conditions (story, back)). Then group statistics (see below) was performed between conditions (story and back).

Group statistics

Group statistical analysis was performed on the data of all 22 participants using non-parametric randomization statistics in FieldTrip (Monte Carlo randomization). Specifically, individual volumetric maps were smoothed with a 10 mm Gaussian kernel and subjected to dependent-samples t-test (story versus back). The null distribution was estimated using 500 randomizations and multiple comparison correction was performed using FDR. Only significant results ($p < 0.05$, FDR-corrected) are reported.

Correlation between speech-brain coherence and top-down index (TDI) at source level

The correlation analysis was designed to test if increased top-down signals correlated with increased coupling between auditory oscillations and the speech envelope. Because our previous analysis had demonstrated that these signals are predominantly directed at left auditory cortex we performed the analysis using transfer entropy to left auditory cortex from the significant voxels shown in Figure 3 A and C. For each of these voxels we correlated the top-down index (TDI) with differential speech-brain coherence (story – back) across the 22 participants. We used non-parametric randomization statistics and corrected for multiple comparisons across voxels using maximum statistics.

Supplemental Results

Lateralization of top-down signals

We performed the main analysis of lateralization on TDI (top-down index) values. The results demonstrate significant lateralization of top-down signals going to left auditory cortex compared to right auditory cortex. These results are presented in the main results section. In addition, we tested lateralization directly on the TE (transfer entropy) values because it allows testing both conditions (story and back) separately. T-test of mean TE values (across significant voxels) for left versus right auditory cortex was significant for the story condition in delta and theta frequency band ($p < 0.05$) but not significant for any frequency band in the back condition. This further supports our conclusion of lateralization to left auditory cortex for the intelligible condition.

Amplitude differences between conditions and possible effects on transfer entropy (TE)

We investigated whether TE differences between conditions could arise from amplitude differences. We used Hilbert transform on band-pass filtered data to separate phase and amplitude information and TE is computed on phase. Thus, in a first approximation the TE measure is independent of amplitude. Still, amplitude differences could affect the reliability of phase estimates. To address this we analyzed the amplitude of delta and theta signals in LAC (left auditory cortex), RAC (right auditory cortex) and higher order areas using the same group statistics that was used for TE data. However, we did not find any significant amplitude difference between story and back conditions for both the delta and theta bands. Thus, we conclude that our TE findings for delta and theta frequency bands are not explained by differences in oscillatory amplitudes. In addition, a recent paper [S13] demonstrates with detailed simulations that phase TE is robust to signal-to-noise changes in realistic conditions.

Supplemental References

S1. Gross, J., Hoogenboom, N., Thut, G., Schyns, P., Panzeri, S., Belin, P., and Garrod, S. (2013). Speech rhythms and multiplexed oscillatory sensory coding in the human brain. PLoS Biol *11*, e1001752.

S2. Pernet, C.R., Wilcox, R., and Rousselet, G.A. (2012). Robust correlation analyses: false positive and power validation using a new open source matlab toolbox. Front Psychol *3*, 606.

S3. Brainard, D.H. (1997). The Psychophysics Toolbox. Spatial vision *10*, 433-436.

S4. Lerner, Y., Honey, C.J., Silbert, L.J., and Hasson, U. (2011). Topographic mapping of a hierarchy of temporal receptive windows using a narrated story. J Neurosci *31*, 2906-2915.

S5. Oostenveld, R., Fries, P., Maris, E., and Schoffelen, J.M. (2011). FieldTrip: Open source software for advanced analysis of MEG, EEG, and invasive electrophysiological data. Computational intelligence and neuroscience *2011*, 156869.

S6. Gross, J., Baillet, S., Barnes, G.R., Henson, R.N., Hillebrand, A., Jensen, O., Jerbi, K., Litvak, V., Maess, B., Oostenveld, R., et al. (2013). Good practice for conducting and reporting MEG research. Neuroimage *65*, 349-363.

S7. Besl, P.J., and McKay, N.D. (1992). A method for registration of 3-D shapes. IEEE T Pattern Anal, 239–256.

S8. Nolte, G. (2003). The magnetic lead field theorem in the quasi-static approximation and its use for magnetoencephalography forward calculation in realistic volume conductors. Physics in medicine and biology *48*, 3637-3652.

S9. Massey, J. (1990). Causality, feedback and directed information. In: Proc. Int. Symp. Information Theory Application (ISITA 1990), 303-305.

S10. Schreiber, T. (2000). Measuring information transfer. Physical review letters *85*, 461-464.

S11. Ince, R.A., Mazzoni, A., Bartels, A., Logothetis, N.K., and Panzeri, S. (2012). A novel test to determine the significance of neural selectivity to single and multiple potentially correlated stimulus features. Journal of neuroscience methods *210*, 49-65.

S12. Panzeri, S., Senatore, R., Montemurro, M.A., and Petersen, R.S. (2007). Correcting for the sampling bias problem in spike train information measures. J Neurophysiol *98*, 1064-1072.

S13. Lobier, M., Siebenhuhner, F., Palva, S., and Palva, J.M. (2014). Phase transfer entropy: a novel phase-based measure for directed connectivity in networks coupled by oscillatory interactions. NeuroImage *85 Pt 2*, 853-872.
