# Supplementary material for: Frontal Top-Down Signals Increase Coupling of Auditory Low-Frequency Oscillations to Continuous Speech in Human Listeners
Source: Curr Biol. 2015 Jun 15;25(12):1649–53. doi: 10.1016/j.cub.2015.04.049 (PMC4503802; doi:10.1016/j.cub.2015.04.049)
Supplement: Document S2. Article plus Supplemental Information [file mmc2.pdf]

Report

Current Biology

Frontal Top-Down Signals Increase Coupling of Auditory Low-Frequency Oscillations to Continuous Speech in Human Listeners

Highlights

- Frontal top-down signals modulate low-frequency oscillations in auditory cortex
- Top-down signals are stronger for left auditory cortex than for right auditory cortex
- Speech-auditory cortex coupling is enhanced as a function of top-down signals

Authors

Hyojin Park, Robin A.A. Ince, Philippe G. Schyns, Gregor Thut, Joachim Gross

Correspondence

hyojin.park@glasgow.ac.uk (H.P.),
joachim.gross@glasgow.ac.uk (J.G.)

In Brief

Park et al. provide new insights into the role of low-frequency oscillations in top-down control during continuous speech processing. They demonstrate causal top-down signals from frontal and motor areas largely directed at left auditory cortex. Speech-auditory cortex coupling is enhanced as a function of stronger top-down signals.

Park et al., 2015, Current Biology *25*, 1649–1653
June 15, 2015 ©2015 The Authors
http://dx.doi.org/10.1016/j.cub.2015.04.049

Current Biology
Report

CellPress

Frontal Top-Down Signals Increase Coupling of Auditory Low-Frequency Oscillations to Continuous Speech in Human Listeners

Hyojin Park,[1,*] Robin A.A. Ince,[1] Philippe G. Schyns,[1] Gregor Thut,[1] and Joachim Gross[1,*]
[1]Institute of Neuroscience and Psychology, University of Glasgow, Glasgow G12 8QB, UK
*Correspondence: hyojin.park@glasgow.ac.uk (H.P.), joachim.gross@glasgow.ac.uk (J.G.)
http://dx.doi.org/10.1016/j.cub.2015.04.049
This is an open access article under the CC BY license (http://creativecommons.org/licenses/by/4.0/).

SUMMARY

Humans show a remarkable ability to understand continuous speech even under adverse listening conditions. This ability critically relies on dynamically updated predictions of incoming sensory information, but exactly how top-down predictions improve speech processing is still unclear. Brain oscillations are a likely mechanism for these top-down predictions [1, 2]. Quasi-rhythmic components in speech are known to entrain low-frequency oscillations in auditory areas [3, 4], and this entrainment increases with intelligibility [5]. We hypothesize that top-down signals from frontal brain areas causally modulate the phase of brain oscillations in auditory cortex. We use magnetoencephalography (MEG) to monitor brain oscillations in 22 participants during continuous speech perception. We characterize prominent spectral components of speech-brain coupling in auditory cortex and use causal connectivity analysis (transfer entropy) to identify the top-down signals driving this coupling more strongly during intelligible speech than during unintelligible speech. We report three main findings. First, frontal and motor cortices significantly modulate the phase of speech-coupled low-frequency oscillations in auditory cortex, and this effect depends on intelligibility of speech. Second, top-down signals are significantly stronger for left auditory cortex than for right auditory cortex. Third, speech-auditory cortex coupling is enhanced as a function of stronger top-down signals. Together, our results suggest that low-frequency brain oscillations play a role in implementing predictive top-down control during continuous speech perception and that top-down control is largely directed at left auditory cortex. This suggests a close relationship between (left-lateralized) speech production areas and the implementation of top-down control in continuous speech perception.

RESULTS

An important aim of our analyses is to test the functional hypothesis that higher-order brain regions influence auditory cortices in a top-down manner to improve the alignment of auditory oscillations with the quasi-rhythmic components of speech (schematically illustrated in Figure 1). We develop our analysis in three steps. First, we demonstrate a top-down directional causal influence of higher-order regions on auditory cortices. Second, we show that this causal influence is primarily lateralized to left auditory cortex. Finally, we show that the functional role of the top-down influence is to improve the speech-brain rhythmic phase alignment.

Our results are based on directional connectivity analysis using transfer entropy (TE). TE is an information theoretic measure that quantifies directed causal effects between time series. We focused our analysis on the phase of low-frequency brain oscillations (delta: 1–3 Hz band; theta: 4–7 Hz band) because they correspond to prosody and syllable rate [3], and they are robustly entrained by continuous speech in auditory cortex (Figure 2, reproducing [4]). This analysis produced volumetric, whole-brain maps where each voxel value represents the strength of directional (top-down) connectivity from this voxel to the left and right auditory cortices, respectively. We computed these two volumetric maps for all 22 participants, two frequency bands (delta, theta), and two experimental conditions (intelligible [story] and unintelligible [back] speech). In the story condition, participants listened to a 7-min-long continuous story. In the back condition, the same story was played backward (see Supplemental Experimental Procedures for details).

First, we identified the brain areas that modulate in a top-down manner the dynamics of auditory phase. We performed false discovery rate (FDR)-corrected group statistics to reveal areas that causally change the phase of auditory delta or theta oscillations significantly more strongly in the story condition than in the back condition (see Figure S1 for opposite contrast, back > story). Figure 3 shows statistical maps of significant TE differences between story and back condition for left and right auditory cortex for delta band (upper panel) and theta band (lower panel). An extensive network of areas showed increased TE in the story condition compared to the back condition to the left auditory cortex (Figures 3A and 3C). For the delta band, these networks comprised right, middle, and inferior temporal gyri, left superior parietal lobule, left inferior frontal gyrus (L-IFG), including Brodmann area (BA) 44, 45, and 47 regions extending to precentral gyrus (BA 6), and right middle and inferior frontal gyri. For the theta band, top-down effects on the left auditory cortex originated in left cuneus, right middle temporal gyrus (BA 37), and left precentral gyrus (BA 4/6). In contrast, only few areas showed increased TE to the right

CrossMark

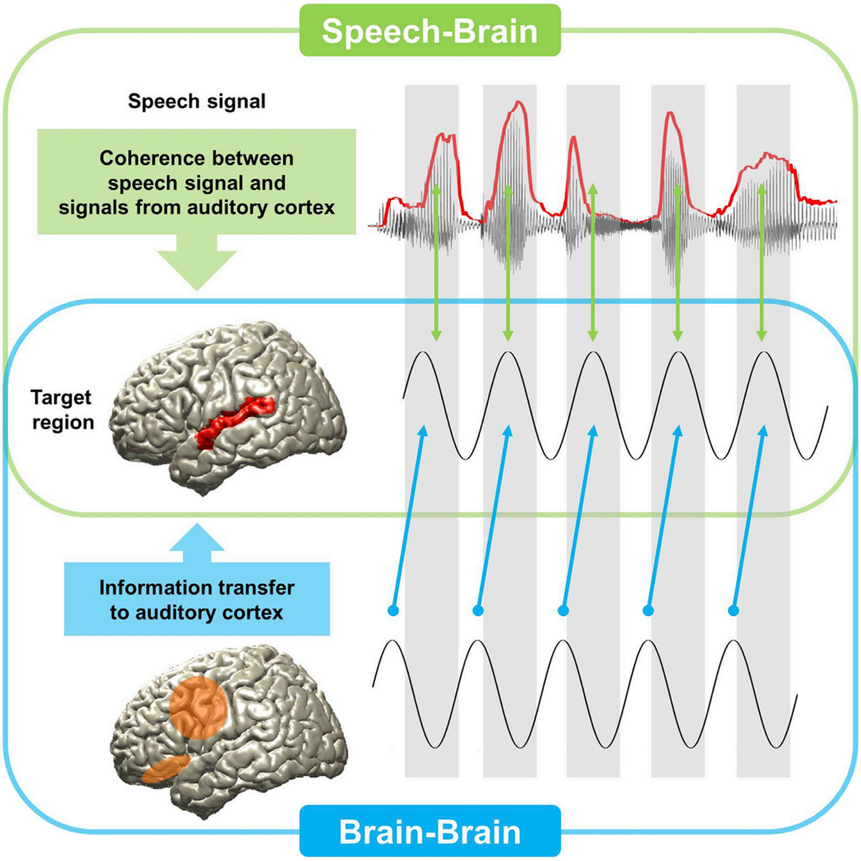

Figure 1. A Schematic Figure of Speech-Brain and Brain-Brain Couplings

Speech-brain coupling (green box): quasi-rhythmic components in speech are known to entrain low-frequency oscillations in auditory areas. This entrainment is evident as coherence between speech envelope and neural activity in auditory cortex (target region). Brain-brain coupling (blue box): we hypothesize that low-frequency oscillations in auditory cortex (target region) are modulated by top-down signals from higher-order areas, thereby changing the gating of speech input.

auditory cortex between story condition and back condition (Figures 3B and 3D). For the delta band, these areas were located in right inferior frontal gyrus and inferior parietal lobule. For the theta band, the right auditory cortex received inputs from areas in right medial frontal gyrus, left inferior parietal lobule, and left middle temporal gyrus. Brain areas and their Montreal Neurological Institute (MNI) coordinates for Figure 3 are shown in Table S1.

The results so far demonstrate that there are higher-order areas whose causal influence on the phase of auditory areas is more pronounced during an intelligible story rather than during an unintelligible story and that this causal influence targets predominantly the left auditory cortex as shown in Figure 3.

Next, we statistically quantified this apparent hemispheric asymmetry. For each participant, we computed a normalized top-down index (TDI) that quantifies the degree to which the identified brain areas (see Figure 3) causally change the phase of left and right auditory cortex differentially for story and back condition. TDI is defined as (TE(story) − TE(back))/(TE(story) + TE(back)). Figure 4 shows the mean TDI for the left and right auditory cortices for the delta (Figure 4A) and theta (Figure 4B) frequency bands computed from the significant areas displayed in Figure 3. TDI was significantly larger than zero for the left auditory cortex in both frequency bands (delta: $t = 4.96$, $p < 0.001$; theta: $t = 4.33$, $p < 0.001$) but not significant for the right auditory cortex (both $p > 0.05$).

In addition, we specifically tested for lateralization of top-down signals to the left versus the right auditory cortex. We statistically compared the mean TDI value across significant voxels (from Figure 3) to the left versus the right auditory cortex. Mean TDI was significantly higher for the left as compared to the right auditory cortex in both the delta ($t = 3.72$, $p < 0.001$) and theta ($t = 3.58$, $p < 0.001$) frequency bands.

These results demonstrate that specific higher-order areas exert a causal top-down effect on low-frequency oscillations in the auditory cortex that is stronger for intelligible speech as compared to unintelligible speech. In addition, we found a significant lateralization of these top-down effects to the left auditory cortex as compared to the right auditory cortex.

Next, we performed further analysis to better characterize top-down signals to left auditory cortex. Delay-specific TDI for left auditory cortex demonstrated that delta TDI is strongest at delays of about 50–60 ms (Figure S2A), whereas theta TDI demonstrates a cyclic modulation at multiple delays (separated by about 40 ms; Figure S2B). We also computed TE time-resolved and centered on ‘‘edges’’ in the continuous speech (following the approach in [4]). Both delta TE and theta TE show increases before and around edge onset (Figures S2C–S2E) in left inferior frontal and precentral gyri.

We further hypothesized that the functional role of the increased top-down effect to the left auditory cortex for the story condition (compared to the back condition) is to increase speech-brain entrainment. This functional hypothesis is based on the notion that predictions about the upcoming speech input will improve the alignment of auditory oscillations to the quasi-rhythmic speech components (such as syllables). To test this hypothesis, we correlated the TDI and differential speech-brain coherence (story − back) across participants. We computed correlations separately for each voxel (from Figure 3) and for delta (Figure 4C) and theta (Figure 4D) bands for TDI to left auditory cortex (threshold at $p < 0.05$, corrected). This analysis revealed significant positive correlations for the delta band that are strongest in left frontal and precentral gyri, indicating that more top-down effects lead to better speech-brain entrainment (Figures 4C, S3A, and S3B). Similarly, for the theta band, the

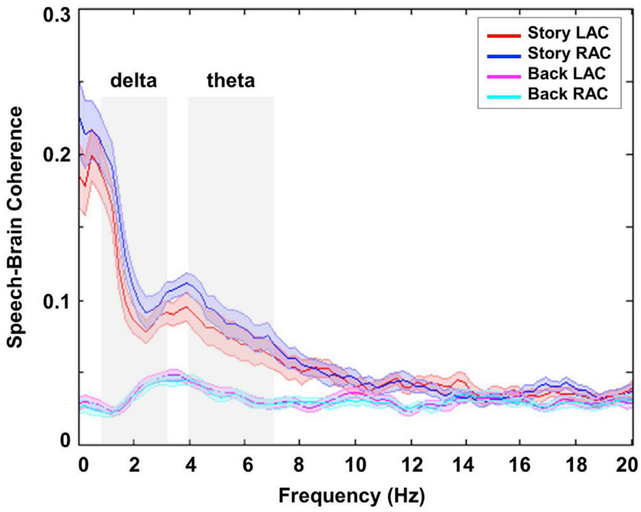

Figure 2. Speech-Brain Coupling
Coherence between speech envelope and low-frequency oscillations (1–20 Hz) in the left auditory cortex (LAC) and right auditory cortex (RAC) in the intelligible speech (story) and unintelligible speech (back) conditions. Low-frequency brain oscillations (delta: 1–3 Hz band; theta: 4–7 Hz band) are entrained by the speech envelope in the intelligible speech (story) condition.

analysis also revealed significant positive correlations with differential speech-brain coherence in left precentral gyrus and posterior temporal areas (Figures 4D, S3C, and S3D).

DISCUSSION

Here, we provide the first direct evidence that top-down signals during speech perception modulate the phase of low-frequency oscillations in the auditory cortex, particularly so in the left auditory cortex.

From a computational perspective, brain oscillations are ideal candidates for the neural implementation of top-down signals from higher-order areas to primary sensory areas [1–3]. During speech processing, they match the frequency of quasi-rhythmic components in speech (such as prosody and syllable rate), are entrained by these speech components, and represent excitability changes of neuronal populations that can be harnessed for gating information flow [6]. Indeed, we and others have recently shown that the phase of cortical oscillations is a likely mechanism for coding and segmentation of continuous speech [3, 4] and visual stimuli [7]. Further support comes from studies demonstrating at least partial spectral dissociation of bottom-up and top-down effects in high-frequency versus low-frequency oscillations, respectively [8, 9].

We observed top-down effects in delta and theta frequency bands. Both bands are functionally distinct in speech processing. Theta oscillations (4–8 Hz) are known to track syllabic rates, whereas delta oscillations (1–3 Hz) are associated with suprasegmental speech components such as intonation, prosody, and phrases [3, 10]. We find stronger top-down effects in delta band compared to theta band, possibly reflecting a preference of top-down signals for longer timescales required to extract

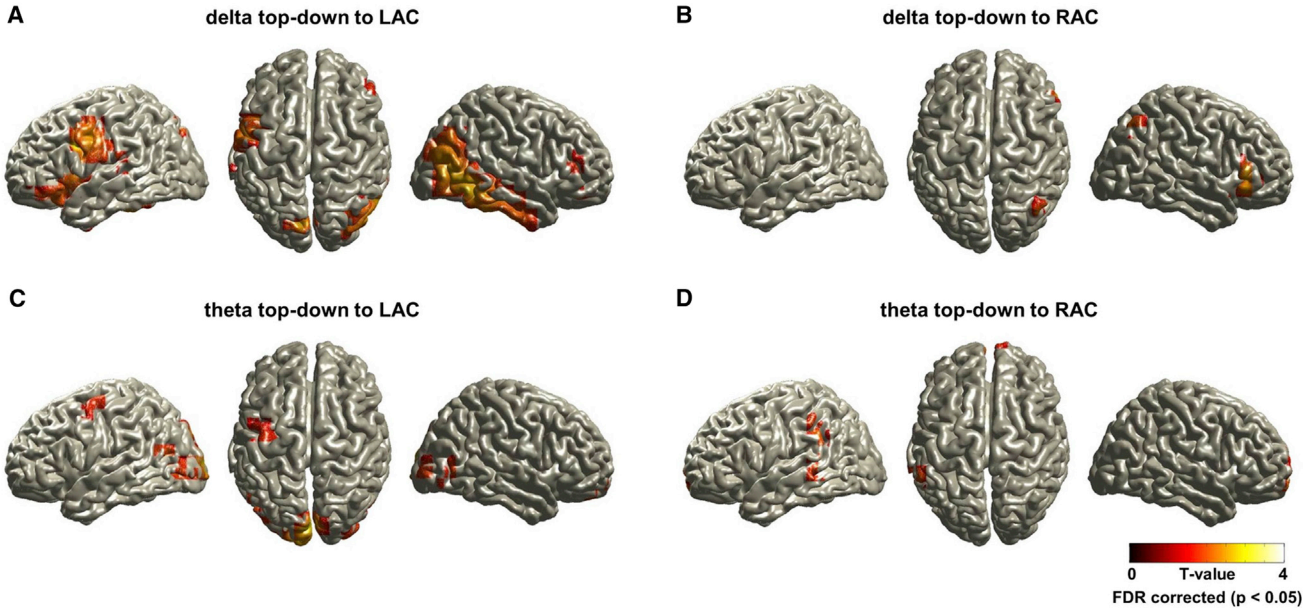

Figure 3. Volumetric Maps of Top-Down TE on Auditory Phase for Delta and Theta Oscillations
Transfer entropy (TE) from each voxel to reference voxels in the LAC and RAC was computed in each condition (story and back), then statistically compared between conditions ($p < 0.05$, corrected for multiple comparisons using FDR). Areas with significantly increased TE in the story condition are shown in (A) delta top-down to LAC, (B) delta top-down to RAC, (C) theta top-down to LAC, and (D) theta top-down to RAC (see Figure S1 for back > story).
(A and C) An extensive network of areas showed increased TE in the story compared to the back condition to LAC. For the delta band (A), the network comprised right middle and inferior temporal gyri, left superior parietal lobule, L-IFG including BA 44, 45, and 47 regions extending to precentral gyrus (BA 6), and right middle and inferior frontal gyri. For the theta band (C), top-down effects on LAC originated in left cuneus, right middle temporal gyrus (BA 37), and left precentral gyrus (BA 4/6).
(B and D) In contrast, only very few areas showed increased TE to RAC between conditions for both delta (B) and theta (D) bands.

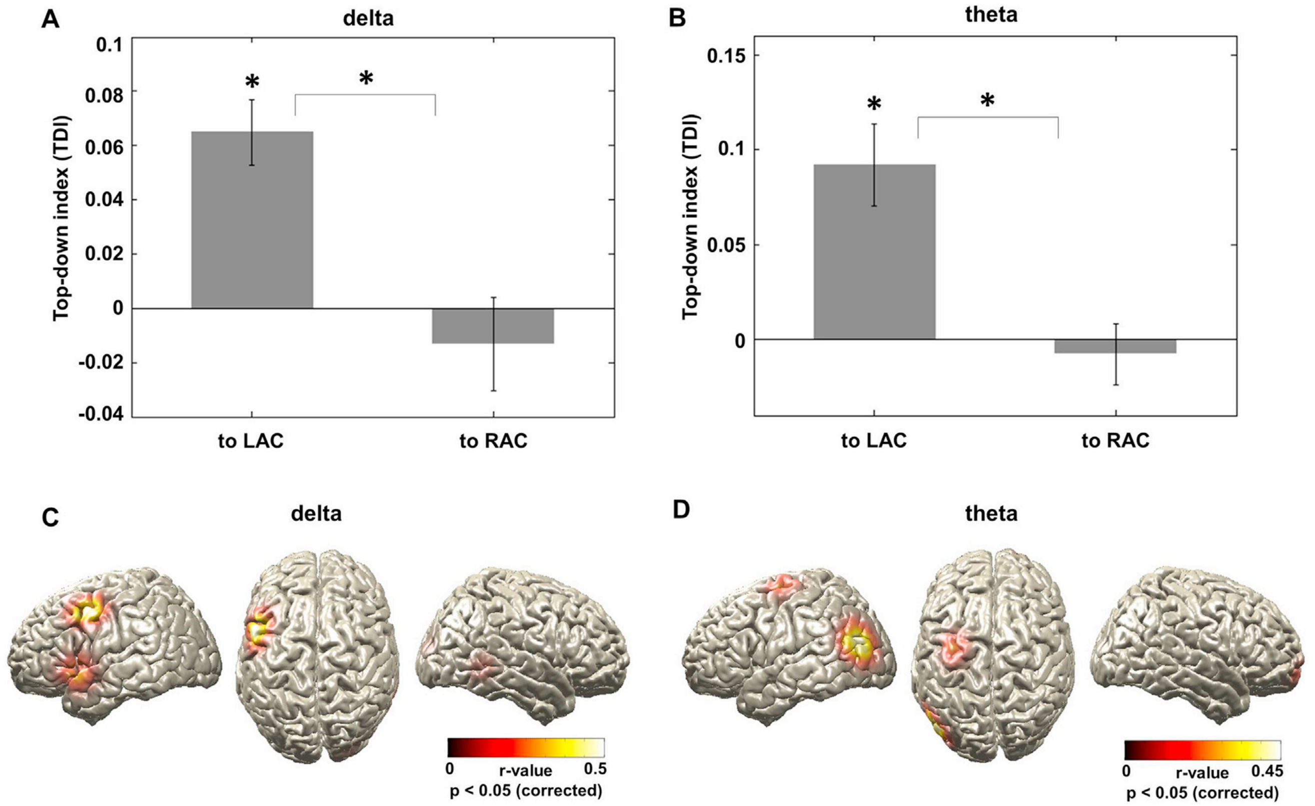

Figure 4. TDI for LAC and RAC and Its Correlation with Differential Speech-Brain Coupling

(A and B) A normalized top-down index (TDI; (TE(story) − TE(back))/(TE(story) + TE(back))) that quantifies the degree to which higher-order brain areas differentially change the phase of LAC and RAC between conditions was computed for each participant. The mean TDI over significant voxels (and SEM) is shown for delta (A) and theta (B) frequency bands.

(C and D) Correlation between TDI and differential speech-brain entrainment. TDI and differential speech-brain coherence (story − back) was correlated across subjects for delta (C) and theta (D) bands ($p < 0.05$, corrected). Results for delta band show significant positive correlations strongest in left frontal and precentral gyri, indicating that more top-down effects lead to better speech-brain entrainment. Results for theta band also show significant positive correlations with speech-brain coherence in left precentral gyrus and posterior temporal areas.

contextual information. Similarly, an fMRI study using the same stimuli identified areas including L-IFG with higher sensitivity to longer timescales that allow extraction of contextual information [11].

Our results imply involvement of left frontal and motor areas in the generation of top-down signals, especially for the delta band. So far, most of our knowledge about likely sources of top-down effects in the context of speech perception comes from fMRI activation studies that use variations of speech intelligibility [12]. These studies point consistently to the L-IFG as a major source of top-down effects on early auditory areas based on increased activation during processing of degraded speech [13–18]. Indeed, anatomical connectivity between L-IFG and auditory cortex is well established in non-human primates [19] and humans [20], and L-IFG has been previously implicated in processes related to the access of mental representations.

Interestingly, recent research converges on the view that speech perception is predominantly bilateral (with different contributions from both hemispheres), whereas speech production is largely left lateralized [21, 22]. The left lateralization of top-down control demonstrated here therefore adds support to recent theories that link speech production and speech perception [23, 24]. This is even more plausible since the generators of top-down signals identified here are compatible with the classical speech production areas. In particular, coordinates of the left precentral gyrus identified here as a source of top-down control are in agreement with previously identified motor areas engaged in speech production [25].

Our causal connectivity analysis demonstrates that the phase of oscillations in the left auditory cortex is modulated more strongly by top-down signals than it is in the right auditory cortex. This is consistent with a recent model (PARLO) postulating lateralization of top-down control to the left hemisphere based on the context-specific modulations of the classical N400 event-related component [26]. Interestingly, the N400 component is generated by the same oscillations (delta/theta) that we have studied here directly and is typically lateralized to left hemisphere [27].

For the delay-specific TDI for theta band (Figure S2B), the periodic modulation of top-down influence at around 25 Hz (one peak every 40 ms) suggests a putative role of the beta band in top-down control, supporting and extending previous reports of a role of beta oscillation in top-down processing [9, 28]. In this study, we focused on delta and theta band because these frequencies correspond directly to prominent components in

speech and show the strongest speech-brain coupling. However, the observed beta modulation warrants further investigation into possible causal effects between frequency bands. Possible top-down control from other frequency bands such as alpha, beta, and gamma could also provide an answer to the dominant peak around 50–60 ms in the delta band delay (Figure S2A). Importantly, future studies might be able to decode the content of these top-down signals.

In summary, we provide direct evidence for the role of low-frequency oscillations in top-down control during speech processing and demonstrate causal top-down signals from higher-order areas more to the left than to the right auditory cortex that improve speech-brain coupling.

SUPPLEMENTAL INFORMATION

Supplemental Information includes Supplemental Results, Supplemental Experimental Procedures, three figures, and one table and can be found with this article online at http://dx.doi.org/10.1016/j.cub.2015.04.049.

AUTHOR CONTRIBUTIONS

J.G. and G.T. conceived and designed the experiments. H.P. and J.G. analyzed the data. R.A.A.I., P.G.S., G.T., and J.G. contributed reagents, materials, and analysis tools. All authors wrote the manuscript.

ACKNOWLEDGMENTS

We thank Nienke Hoogenboom for recording the data. This study was supported by the Wellcome Trust (098433, 098434) and by the ESRC and MRC (RES-060-25-0010). The funders had no role in study design, data collection and analysis, decision to publish, or preparation of the manuscript.

Received: January 21, 2015
Revised: March 23, 2015
Accepted: April 23, 2015
Published: May 28, 2015

REFERENCES

1. Arnal, L.H., and Giraud, A.L. (2012). Cortical oscillations and sensory predictions. Trends Cogn. Sci. *16*, 390–398.
2. Engel, A.K., Fries, P., and Singer, W. (2001). Dynamic predictions: oscillations and synchrony in top-down processing. Nat. Rev. Neurosci. *2*, 704–716.
3. Giraud, A.L., and Poeppel, D. (2012). Cortical oscillations and speech processing: emerging computational principles and operations. Nat. Neurosci. *15*, 511–517.
4. Gross, J., Hoogenboom, N., Thut, G., Schyns, P., Panzeri, S., Belin, P., and Garrod, S. (2013). Speech rhythms and multiplexed oscillatory sensory coding in the human brain. PLoS Biol. *11*, e1001752.
5. Peelle, J.E., Gross, J., and Davis, M.H. (2013). Phase-locked responses to speech in human auditory cortex are enhanced during comprehension. Cereb. Cortex *23*, 1378–1387.
6. Schroeder, C.E., Lakatos, P., Kajikawa, Y., Partan, S., and Puce, A. (2008). Neuronal oscillations and visual amplification of speech. Trends Cogn. Sci. *12*, 106–113.
7. Schyns, P.G., Thut, G., and Gross, J. (2011). Cracking the code of oscillatory activity. PLoS Biol. *9*, e1001064.
8. Buffalo, E.A., Fries, P., Landman, R., Buschman, T.J., and Desimone, R. (2011). Laminar differences in gamma and alpha coherence in the ventral stream. Proc. Natl. Acad. Sci. USA *108*, 11262–11267.
9. Fontolan, L., Morillon, B., Liegeois-Chauvel, C., and Giraud, A.L. (2014). The contribution of frequency-specific activity to hierarchical information processing in the human auditory cortex. Nat. Commun. *5*, 4694.
10. Cogan, G.B., and Poeppel, D. (2011). A mutual information analysis of neural coding of speech by low-frequency MEG phase information. J. Neurophysiol. *106*, 554–563.
11. Lerner, Y., Honey, C.J., Silbert, L.J., and Hasson, U. (2011). Topographic mapping of a hierarchy of temporal receptive windows using a narrated story. J. Neurosci. *31*, 2906–2915.
12. Scott, S.K., and McGettigan, C. (2013). The neural processing of masked speech. Hear. Res. *303*, 58–66.
13. Binder, J.R., Liebenthal, E., Possing, E.T., Medler, D.A., and Ward, B.D. (2004). Neural correlates of sensory and decision processes in auditory object identification. Nat. Neurosci. *7*, 295–301.
14. Davis, M.H., and Johnsrude, I.S. (2003). Hierarchical processing in spoken language comprehension. J. Neurosci. *23*, 3423–3431.
15. Davis, M.H., and Johnsrude, I.S. (2007). Hearing speech sounds: top-down influences on the interface between audition and speech perception. Hear. Res. *229*, 132–147.
16. Giraud, A.L., Kell, C., Thierfelder, C., Sterzer, P., Russ, M.O., Preibisch, C., and Kleinschmidt, A. (2004). Contributions of sensory input, auditory search and verbal comprehension to cortical activity during speech processing. Cereb. Cortex *14*, 247–255.
17. Hervais-Adelman, A.G., Carlyon, R.P., Johnsrude, I.S., and Davis, M.H. (2012). Brain regions recruited for the effortful comprehension of noise-vocoded words. Lang. Cogn. Process. *27*, 1145–1166.
18. Zekveld, A.A., Heslenfeld, D.J., Festen, J.M., and Schoonhoven, R. (2006). Top-down and bottom-up processes in speech comprehension. Neuroimage *32*, 1826–1836.
19. Hackett, T.A., Stepniewska, I., and Kaas, J.H. (1999). Prefrontal connections of the parabelt auditory cortex in macaque monkeys. Brain Res. *817*, 45–58.
20. Saur, D., Kreher, B.W., Schnell, S., Kümmerer, D., Kellmeyer, P., Vry, M.S., Umarova, R., Musso, M., Glauche, V., Abel, S., et al. (2008). Ventral and dorsal pathways for language. Proc. Natl. Acad. Sci. USA *105*, 18035–18040.
21. Hickok, G. (2012). Computational neuroanatomy of speech production. Nat. Rev. Neurosci. *13*, 135–145.
22. Jung-Beeman, M. (2005). Bilateral brain processes for comprehending natural language. Trends Cogn. Sci. *9*, 512–518.
23. Sohoglu, E., Peelle, J.E., Carlyon, R.P., and Davis, M.H. (2012). Predictive top-down integration of prior knowledge during speech perception. J. Neurosci. *32*, 8443–8453.
24. Pickering, M.J., and Garrod, S. (2007). Do people use language production to make predictions during comprehension? Trends Cogn. Sci. *11*, 105–110.
25. Wilson, S.M., Saygin, A.P., Sereno, M.I., and Iacoboni, M. (2004). Listening to speech activates motor areas involved in speech production. Nat. Neurosci. *7*, 701–702.
26. Federmeier, K.D. (2007). Thinking ahead: the role and roots of prediction in language comprehension. Psychophysiology *44*, 491–505.
27. Pylkkänen, L., and Marantz, A. (2003). Tracking the time course of word recognition with MEG. Trends Cogn. Sci. *7*, 187–189.
28. Bastos, A.M., Vezoli, J., Bosman, C.A., Schoffelen, J.M., Oostenveld, R., Dowdall, J.R., De Weerd, P., Kennedy, H., and Fries, P. (2015). Visual areas exert feedforward and feedback influences through distinct frequency channels. Neuron *85*, 390–401.

Current Biology
Supplemental Information

Frontal Top-Down Signals Increase Coupling of Auditory Low-Frequency Oscillations to Continuous Speech in Human Listeners

Hyojin Park, Robin A.A. Ince, Philippe G. Schyns, Gregor Thut, and Joachim Gross

Supplemental Figures and Legends

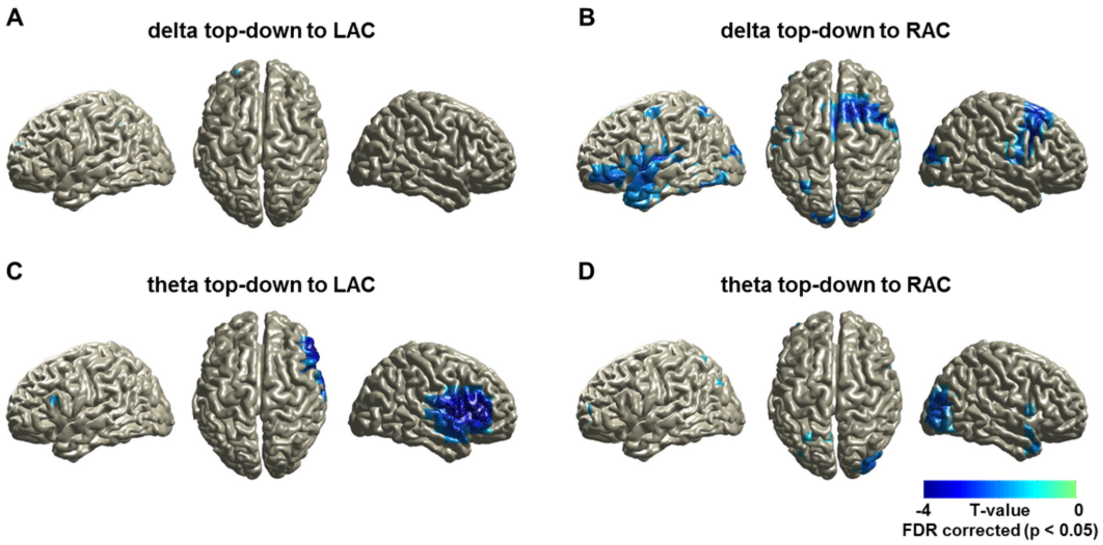

Figure S1 related to Figure 3. Transfer entropy (TE) for back condition compared to story condition

We performed group statistics on TE values for the contrast back > story condition for delta and theta bands ($p < 0.05$, FDR-corrected). Interestingly, for the delta band, top-down effects that are stronger in back compared to story condition are largely directed at right auditory cortex (Figure S1 B). In contrast, the statistical contrast story > back shows a significant lateralization towards left auditory cortex (Figure 3, 4). The most pronounced effect in the theta band is a stronger top-down effect in back compared to story condition from right temporal/frontal areas to left auditory cortex (Figure S1 C).

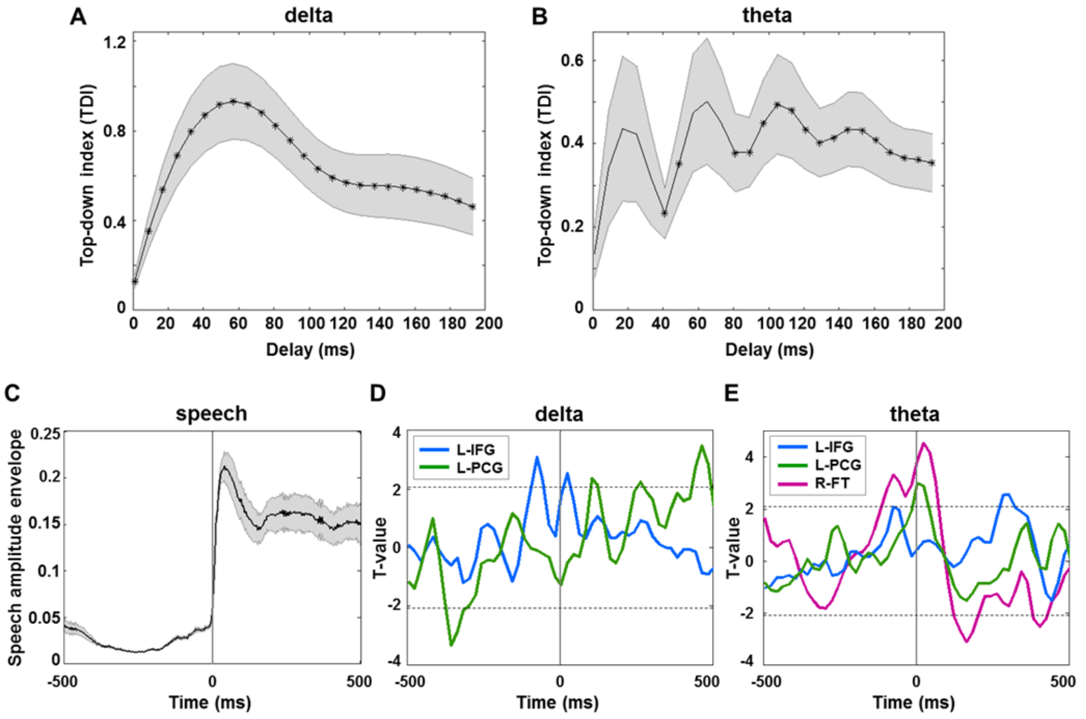

Figure S2 related to Figure 4 A and B. Top-down index (TDI) to left auditory cortex for each delay and temporal dynamics of TE time-locked to speech edges

(A and B): Top-down index (TDI) for each delay. Figure 3 shows TE averaged across 25 delays (in 8 ms steps up to 200 ms). In addition, we show here TDI to left auditory cortex for individual delays averaged over the significant voxels from story versus back condition. We compared TDI against zero at each time point (asterisk: $p < 0.05$, corrected). Delay-specific TDI for delta band (A) peaks at a delay of about 50-60 ms. This seems to be a characteristic delay where the difference between TE for story and back condition is maximal. Further investigations are required to clarify the content of this signal. Theta band (B) shows a cyclic modulation with several peaks at delays separated by about 40 ms corresponding to a frequency in the beta band. Significant differences between conditions can also be seen at the troughs of TDI modulation and seem to be caused by timing differences of TE between story and back condition.

(C, D, and E): Temporal dynamics of TE time-locked to speech edges. We have followed the approach from our previous paper [S1] and performed the transfer entropy computation in a time-resolved manner locked to the onset of edges. Edges were defined (consistent with our previous paper) as short breaks in the continuous speech followed by significant increases in the amplitude envelope. Mean and variance of time-locked speech amplitude envelope is shown in C. Time axis (0 ms; gray vertical line) is centered on the latency at the start of the rising amplitude envelope. We computed time-resolved transfer entropy (TE) to LAC (left auditory cortex) for all voxels that showed a significant condition effect as shown in Figure 3 (A, C). Across the 22 participants we computed T-values of the condition effect (story versus back) for each time point between -500 ms and 500 ms. For each brain area where the T-value was significant at $p < 0.005$ (uncorrected) for at least one time point we extracted the time series of T-values. T-value of significance level ($t = \pm 2.08$ at $p < 0.05$) is shown by horizontal dashed lines. Interestingly, different temporal pattern can be seen. In the delta frequency band (D), T-values for left IFG (L-IFG; blue) peak just before and after the onset and decrease after that (with a significantly negative slope). In contrast, T-values for left precentral gyrus (L-PCG; green) show a gradual increase (with a significant positive slope). In the theta band (E), the strongest effect is observed in right fronto-temporal areas (R-FT; purple). The T-values increase towards edge onset (reflecting significantly stronger TE for story versus back) and change sign after edge onset (reflecting significantly weaker TE for story versus back). Left IFG (L-IFG; blue) and left precentral gyrus (L-PCG; green) show strongest T-values just before or around the edge onset.

These results suggest that different areas could mediate different predictions. Those showing strongest effects around or before edge onset likely transfer predictions about the temporal onset of edges and possibly about expected syllables or phonemes although this remains speculation at this point. It should be noted that even TE values computed for time 0 ms will be based on the past 200 ms of data due to the computation of TE. Those areas showing an increase after edge onset will likely express predictions based on the incoming sensory information. For example, the difference in time between the peaks for the two regions shown in the delta band (D) suggests that the frontal region could be more involved in the prediction of upcoming speech components

(e.g. timing and content of speech onset) whereas motor region could be more involved in continuously improving predictions (after stimulus onset) based on incoming information, yet this remains speculative. Unfortunately it is difficult to track TE values for longer periods of time (e.g. seconds) because of the variability in the stimuli following an edge. Some edges will be followed by longer periods of ‘continuous’ speech whereas others might show another edge after a short period of time. In summary, this analysis suggests that different brain areas mediate different types of predictions.

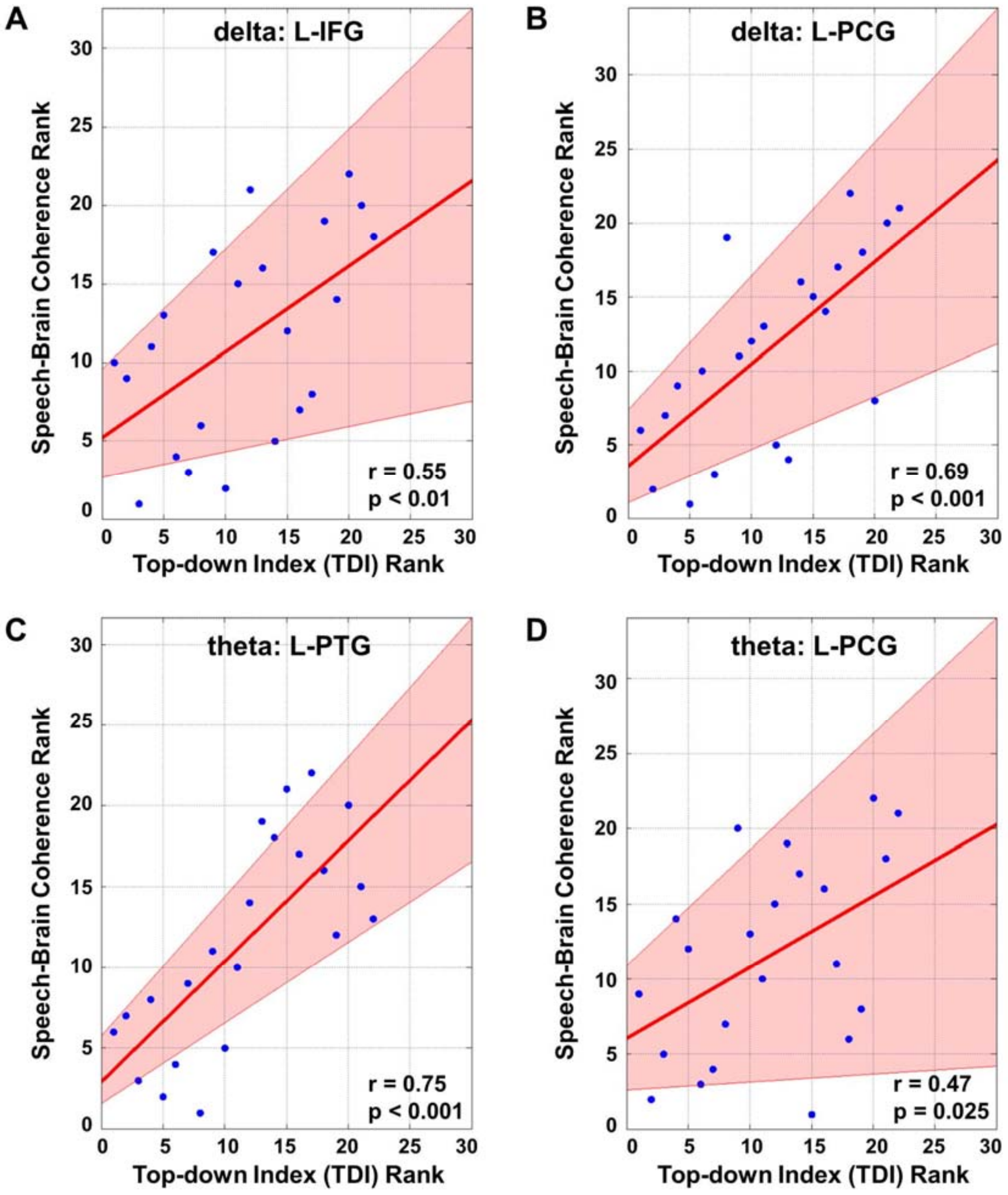

Figure S3 related to Figure 4 C and D. Correlations between top-down index (TDI) and speech-brain coupling at maximum sources

To confirm the correlations shown in Figure 4 C (delta) and D (theta), we show here the scatter plots of correlations for each significant area for delta and theta bands from Figure 4 C and D. To make sure that effects are not dominated by outliers we computed robust correlations using an

open-source toolbox [S2]. Specifically, we computed Spearman rank correlations with the 95% bootstrapped confidence interval (pink shaded areas) computed from 1000 iterations. All reported correlations are significant also based on bootstrap confidence interval.

(A) L-IFG (left inferior frontal gyrus) in the delta band: $r = 0.55$, $p < 0.01$

(B) L-PCG (left precentral gyrus) in the delta band: $r = 0.69$, $p < 0.001$

(C) L-PTG (left posterior temporal gyrus) in the theta band: $r = 0.75$, $p < 0.001$

(D) L-PCG (left precentral gyrus) in the theta band: $r = 0.47$, $p = 0.025$

Supplemental Table

Frequency band	MNI coordinates			Brain regions	L/R	BA
	**To Left Auditory Cortex (LAC)**					
	**x**	**y**	**z**			
**delta**	-52	-2	34	Inferior Frontal Gyrus (Pars Opercularis) /Precentral gyrus	L	44/6
	-42	31	-17	Inferior Frontal Gyrus	L	45/47
	-10	-80	40	Superior Parietal Lobule/Precuneus	L	7
	68	-58	-6	Middle/Inferior Temporal Gyrus	R	21
	52	39	15	Middle/Inferior Frontal Gyrus (triangular part)	R	46
**theta**	-45	-11	63	Precentral Gyrus	L	4/6
	0	-90	30	Cuneus	L	19
	60	-71	5	Middle Temporal Gyrus	R	37

Frequency band	MNI coordinates			Brain regions	L/R	BA
	**To Right Auditory Cortex (RAC)**					
	**x**	**y**	**z**			
**delta**	65	30	4	Inferior Frontal Gyrus (triangular part)	R	45
	50	-69	53	Inferior Parietal Lobule/Angular Gyrus	R	40
**theta**	-66	-44	1	Middle Temporal Gyrus	L	22
	-65	-46	36	Inferior Parietal Lobule/Supramarginal Gyrus	L	40
	5	70	-10	Medial Frontal Gyrus (orbital part)	R	11
	2	-61	-50	Cerebellum 9/Inferior Semi-Lunar Lobule	R	

Table S1 related to Figure 3. Brain areas observed in top-down transfer entropy (TE) to LAC and RAC

Brain areas and their MNI coordinates as well as Brodmann Areas (BA) for Figure 3 for both delta and theta bands are displayed.

Supplemental Experimental Procedures

Participants

22 healthy, right-handed volunteers participated in the study (11 males; age range 19–44 years, mean 27 years). All participants provided informed written consent and received monetary compensation for their participation. The study was approved by the local ethics committee (University of Glasgow, Faculty of Information and Mathematical Sciences) and conducted in conformity with the Declaration of Helsinki.

Stimulus

The main stimulus consisted of a recording of a 7-minute real-life story ("Pie-man," told by Jim O'Grady at "The Moth" storytelling event, New York). The story was presented binaurally via a sound pressure transducer through two 5 meters long plastic tubes terminating in plastic insert earpieces. Presentation was controlled with Psychtoolbox [S3] under MATLAB (MathWorks, Natick, MA). In addition to one standard presentation of the story (story condition), individuals also listened to the backward played story (back condition). Eye fixation was maintained throughout the experiment. Experimental conditions were recorded in randomized order. Stimuli have been previously used in an fMRI study [S4].

Data recording

MEG recordings were obtained with a 248-magnetometers whole-head MEG system (MAGNES 3600 WH, 4-D Neuroimaging) at 1017 Hz sampling rate. The analysis of the MEG signal was

performed using the FieldTrip toolbox [S5] (http://fieldtrip.fcdonders.nl) and in-house MATLAB code according to recently published guidelines [S6]. Another analysis of the same data has been published recently [S1].

MEG-MRI co-registration

T1-weighted structural magnetic resonance images (MRIs) of each participant were co-registered to the MEG coordinate system using a semi-automatic procedure. Anatomical landmarks (nasion, left and right pre-auricular points) were manually identified in the individual's MRI. Initial alignment of both coordinate systems was based on these three points. Subsequently, numerical optimization was achieved by using the ICP algorithm [S7]. All region-of-interest analysis for the auditory cortex is based on the mean effect of all voxels in BA 41. BA 41 voxels were the same for all participants and defined anatomically from the MNI template brain.

Source localization

Individual head models were created from anatomical MRIs using segmentation routines in FieldTrip/SPM5. Leadfield computation was based on a single shell volume conductor model [S8] using a 10 mm grid defined on the template (MNI) brain. The template grid was transformed into individual head space by linear spatial transformation. Cross-spectral density matrices were computed using Fast Fourier Transform on 1-s segments of data after applying Hanning window.

For each voxel, we computed frequency-specific spatial filters for delta (1–3 Hz) and theta (4–7 Hz) frequency bands in the dominant dipole orientation. First, we computed the covariance matrix over the full broad-band 7-minute data set to compute LCMV filters for each voxel using 7 % regularization. These time series were subjected to band-pass filtering (4th order Butterworth filter, forward and reverse). We used the SVD approach to estimate the dominant orientation

independently for each voxel. Bandpass filtered data were projected through the filter to obtain band-limited time-series for each voxel, both frequency bands (delta, theta) and both conditions (story, back). Finally, Hilbert transform was applied to compute time series of instantaneous phase.

Transfer Entropy Analysis

Transfer Entropy (TE), also known as Directed Information [S9, S10], quantifies directed statistical dependencies between two signals. Specifically, TE from signal *X* to signal *Y* quantifies to what extent knowledge of *X* reduces uncertainty in predicting the future of *Y* over and above what could be predicted from knowledge of the past of *Y* alone. TE is conceptually similar to Granger causality as it infers causal relationships from time-lagged predictability.

To determine the TE between two voxels *X* and *Y* during a particular condition (story or back), we first quantized the phase values obtained across all time points during the stimulus presentation, separately for each voxel. We used 4 bins and the bin boundaries were chosen so that the bins were equally occupied (i.e. bins represent quartiles of the distribution of phase values between $-\pi$ and π). For a specific delay *d*, we calculated TE from *X* to *Y* from the following equation:

$$TE_d(X \rightarrow Y) = CMI\left(X_d;Y \mid Y_d\right)$$
$$= H(X_d,Y_d) + H(Y,Y_d) - H(X_d,Y,Y_d) - H(Y_d)$$

Where *CMI* is conditional mutual information, *H* represents entropy and the suffix *d* represents that signal is delayed with respect to the target signal *Y* by *d* milliseconds (i.e. considers that signal *d* milliseconds prior to *Y*). We computed entropy terms from the standard formula:

$$H(Y,Y_d) = \sum\nolimits_{a,b=1}^{4} p_{Y,Y_d}\left(a,b\right) \log_2 p_{Y,Y_d}\left(a,b\right)$$

Where the joint distribution $p_{Y,Y_d}(a,b)$ is obtained from the multinomial maximum likelihood estimate obtained over time points:

$$p_{Y,Y_d}(a,b) = \sum_{t=d}^{Nt} \frac{\delta_a(Y(t))\delta_b(Y(t-d))}{Nt}$$

With $\delta_a(Y(t))$ a Kronecker delta function taking the value 1 if the binned phase value at *Y(t)* is quantile *a* and 0 otherwise.

No bias correction was applied since we performed statistical analysis on contrasts between conditions [S11]. For each calculation, the same number of bins was used and the same number of time points was available. To first order, the bias of mutual information depends only on these parameters [S12], so should be similar across the conditions being compared. Bias correction methods reduce bias but increase the variance of the estimator, so in this case comparisons between calculations with the same bias are better made with uncorrected estimates.

We selected all voxels in the left and right primary auditory cortices (BA 41) as reference voxels. For each reference voxel, TE was computed, as described above, from the binned instantaneous phase of all other voxels in the brain to that reference voxel. The calculation was repeated for 25 different delays, from 8 ms to 200 ms (8 ms steps).

These computations were performed for each participant, both frequency bands and both conditions (story, back). Subsequently, individual TE maps were averaged across delays and across reference voxels in the left and the right auditory cortex (BA 41) separately. Since we were interested in top-down effects on phase dynamics in the left and the right BA 41, we analyzed only the TE corresponding to causal effects from other voxels on these two reference regions. This resulted in eight TE maps per participant (two frequency bands (delta, theta), two reference regions (left and right auditory cortices) and two conditions (story, back)). Then group statistics (see below) was performed between conditions (story and back).

Group statistics

Group statistical analysis was performed on the data of all 22 participants using non-parametric randomization statistics in FieldTrip (Monte Carlo randomization). Specifically, individual volumetric maps were smoothed with a 10 mm Gaussian kernel and subjected to dependent-samples t-test (story versus back). The null distribution was estimated using 500 randomizations and multiple comparison correction was performed using FDR. Only significant results ($p < 0.05$, FDR-corrected) are reported.

Correlation between speech-brain coherence and top-down index (TDI) at source level

The correlation analysis was designed to test if increased top-down signals correlated with increased coupling between auditory oscillations and the speech envelope. Because our previous analysis had demonstrated that these signals are predominantly directed at left auditory cortex we performed the analysis using transfer entropy to left auditory cortex from the significant voxels shown in Figure 3 A and C. For each of these voxels we correlated the top-down index (TDI) with differential speech-brain coherence (story – back) across the 22 participants. We used non-parametric randomization statistics and corrected for multiple comparisons across voxels using maximum statistics.

Supplemental Results

Lateralization of top-down signals

We performed the main analysis of lateralization on TDI (top-down index) values. The results demonstrate significant lateralization of top-down signals going to left auditory cortex compared to right auditory cortex. These results are presented in the main results section. In addition, we tested lateralization directly on the TE (transfer entropy) values because it allows testing both conditions (story and back) separately. T-test of mean TE values (across significant voxels) for left versus right auditory cortex was significant for the story condition in delta and theta frequency band ($p < 0.05$) but not significant for any frequency band in the back condition. This further supports our conclusion of lateralization to left auditory cortex for the intelligible condition.

Amplitude differences between conditions and possible effects on transfer entropy (TE)

We investigated whether TE differences between conditions could arise from amplitude differences. We used Hilbert transform on band-pass filtered data to separate phase and amplitude information and TE is computed on phase. Thus, in a first approximation the TE measure is independent of amplitude. Still, amplitude differences could affect the reliability of phase estimates. To address this we analyzed the amplitude of delta and theta signals in LAC (left auditory cortex), RAC (right auditory cortex) and higher order areas using the same group statistics that was used for TE data. However, we did not find any significant amplitude difference between story and back conditions for both the delta and theta bands. Thus, we conclude that our TE findings for delta and theta frequency bands are not explained by differences in oscillatory amplitudes. In addition, a recent paper [S13] demonstrates with detailed simulations that phase TE is robust to signal-to-noise changes in realistic conditions.

Supplemental References

S1. Gross, J., Hoogenboom, N., Thut, G., Schyns, P., Panzeri, S., Belin, P., and Garrod, S. (2013). Speech rhythms and multiplexed oscillatory sensory coding in the human brain. PLoS Biol *11*, e1001752.

S2. Pernet, C.R., Wilcox, R., and Rousselet, G.A. (2012). Robust correlation analyses: false positive and power validation using a new open source matlab toolbox. Front Psychol *3*, 606.

S3. Brainard, D.H. (1997). The Psychophysics Toolbox. Spatial vision *10*, 433-436.

S4. Lerner, Y., Honey, C.J., Silbert, L.J., and Hasson, U. (2011). Topographic mapping of a hierarchy of temporal receptive windows using a narrated story. J Neurosci *31*, 2906-2915.

S5. Oostenveld, R., Fries, P., Maris, E., and Schoffelen, J.M. (2011). FieldTrip: Open source software for advanced analysis of MEG, EEG, and invasive electrophysiological data. Computational intelligence and neuroscience *2011*, 156869.

S6. Gross, J., Baillet, S., Barnes, G.R., Henson, R.N., Hillebrand, A., Jensen, O., Jerbi, K., Litvak, V., Maess, B., Oostenveld, R., et al. (2013). Good practice for conducting and reporting MEG research. Neuroimage *65*, 349-363.

S7. Besl, P.J., and McKay, N.D. (1992). A method for registration of 3-D shapes. IEEE T Pattern Anal, 239–256.

S8. Nolte, G. (2003). The magnetic lead field theorem in the quasi-static approximation and its use for magnetoencephalography forward calculation in realistic volume conductors. Physics in medicine and biology *48*, 3637-3652.

S9. Massey, J. (1990). Causality, feedback and directed information. In: Proc. Int. Symp. Information Theory Application (ISITA 1990), 303-305.

S10. Schreiber, T. (2000). Measuring information transfer. Physical review letters *85*, 461-464.

S11. Ince, R.A., Mazzoni, A., Bartels, A., Logothetis, N.K., and Panzeri, S. (2012). A novel test to determine the significance of neural selectivity to single and multiple potentially correlated stimulus features. Journal of neuroscience methods *210*, 49-65.

S12. Panzeri, S., Senatore, R., Montemurro, M.A., and Petersen, R.S. (2007). Correcting for the sampling bias problem in spike train information measures. J Neurophysiol *98*, 1064-1072.

S13. Lobier, M., Siebenhuhner, F., Palva, S., and Palva, J.M. (2014). Phase transfer entropy: a novel phase-based measure for directed connectivity in networks coupled by oscillatory interactions. NeuroImage *85 Pt 2*, 853-872.
